# Chemogenomics for steroid hormone receptors (NR3)

**DOI:** 10.1038/s42004-025-01427-z

**Published:** 2025-02-03

**Authors:** Espen Schallmayer, Laura Isigkeit, Lewis Elson, Susanne Müller, Stefan Knapp, Julian A. Marschner, Daniel Merk

**Affiliations:** 1 Institute of Pharmaceutical Chemistry, Goethe-Universität Frankfurt, Frankfurt, Germany; 2 Department of Pharmacy, Ludwig-Maximilians-Universität München, Munich, Germany

**Keywords:** Target identification, Chemical libraries, Target validation, Target identification

## Abstract

The nine human NR3 nuclear receptors translate steroid hormone signals in transcriptomic responses and operate multiple highly important processes ranging from development over reproductive tissue function to inflammatory and metabolic homeostasis. Although several NR3 ligands such as glucocorticoids are invaluable drugs, this family is only partially explored, for example, in autoimmune diseases and neurodegeneration, but may hold therapeutic potential in new areas. Here we report a chemogenomics (CG) library to reveal elusive effects of NR3 receptor modulation in phenotypic settings. 34 highly annotated and chemically diverse ligands covering all NR3 receptors were selected considering complementary modes of action and activity, selectivity and lack of toxicity. Endoplasmic reticulum stress resolving effects of N3 CG subsets in proof-of-concept application validate suitability of the set to connect phenotypic outcomes with targets and to explore NR3 receptors from a translational perspective.

## Abstract (web summary)

Human NR3 nuclear receptors translate steroid hormone signals in transcriptomic responses and serve as invaluable drug targets, however, their therapeutic potential remains underexplored. Here, the authors report a chemogenomics library of NR3 ligands to reveal elusive effects of NR3 receptor modulation in phenotypic settings, revealing potential NR3-mediated effects in endoplasmic reticulum stress and providing proof-of-concept for its suitability to correlate phenotypic outcomes with molecular targets.

## Introduction

**Fig. 1:**
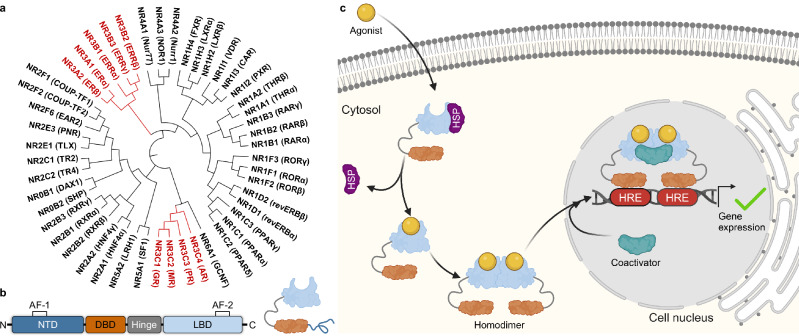
Structure and function of NR3 receptors. **a** Phylogenetic tree of the NR family comprising 48 members in human. NR3 receptors are labeled in red. **b** The archetypal domain structure of NRs is composed of an unordered N-terminal domain (NTD; dark blue), containing the ligand-independent activation function 1 (AF-1), a DNA binding domain (DBD; orange) with two zinc finger motifs for binding to hormone response elements (HREs) on the DNA, a flexible hinge region (grey), and a ligand binding domain (LBD; light blue) with the ligand-dependent activation function 2 (AF-2). **c** General activation mechanism of NR3 receptors. Colors corresponding to modular domain structure in (**b**). Before agonist binding (yellow), most NR3 receptors are located in the cytosol bound to heat shock proteins (HSPs; purple) preventing diffusion of the receptor into the nucleus. Agonist binding leads to conformational changes, release of the HSP, and receptor homodimerization. The resulting dimer diffuses to the nucleus, binds the corresponding HRE (red) on the DNA (dark grey), and recruits coactivators (cyan) to initiate gene transcription. Estrogen receptors (ER; NR3A) and progesterone receptor (PR; NR3C3) can also be DNA-bound in the nucleus independently of ligand binding and thus deviate from the general mechanism^72–75^. Created in BioRender. Merk, D. (2025) https://BioRender.com/a87p902.

As endocrine signal molecules, steroid hormones operate a multitude of physiological processes with major phenotypic impact. The stress hormone cortisol, for example, widely affects glucose metabolism and inflammation^1,2^, aldosterone maintains cardiovascular homeostasis^3^, and the sex steroids (estrogens, androgens and gestagens) regulate biological sex differentiation and reproduction^4^. While several steroid hormones exhibit also rapid non-genomic actions^5,6^, the transcriptional effects of corticosteroids and sex steroids are mediated via the nuclear hormone receptors of the NR3 family^7,8^ (Fig. 1). NR3 receptors act as ligand-sensing transcription factors and comprise nine proteins with common architecture^9,10^ but substantially different ligand recognition.

The pharmacological importance and therapeutic potential of NR3 receptors mediating steroid hormone activities has long been recognized. Invaluable drugs like glucocorticoids for anti-inflammatory treatment^11^ and estrogens for contraception^12^ have been discovered decades ago and still have remarkable importance. Additionally, targeting of sex steroid receptors (ER, NR3A; AR, NR3C4) is a widely used approach in treating hormone-dependent cancer^13,14^ and mineralocorticoid receptor (MR, NR3C2) antagonists are key drugs in cardiovascular diseases^15^. Additionally, a growing body of research supports importance of steroid hormones and their receptors in multiple other organs and pathologies including, for example, immunology and autoimmune diseases^16,17^, metabolic dysfunction^18,19^, and neurodegeneration^20,21^. Further exploration of NR3 receptors from a translational perspective may, therefore, open new therapeutic avenues, and sets of highly annotated NR3 ligands for chemogenomics (CG)^22–24^ could serve as a valuable tool to reveal unprecedented roles of the steroid hormone receptors.

CG is an emerging approach to target identification and validation employing optimized libraries of extensively characterized bioactive molecules for phenotypic screening in disease-relevant in vitro models^22–24^. Only few dedicated CG compound sets have been developed for this purpose, e.g., for kinases^25^, and NR1 nuclear hormone receptors^26^, but a vast collection of optimized and broadly characterized bioactive molecules for hundreds of targets^27^ is available from medicinal chemistry programs to assemble further CG sets and promote translational target identification.

Here we report the rational compilation of a CG library for the NR3 nuclear hormone receptors, its comprehensive characterization, and its application in phenotypic settings. Rigorous filtering of available NR3 modulators and optimization of their combination for chemical diversity and non-overlapping selectivity profiles resulted in a set of 34 compounds fully covering the nine NR3 receptors. Proof-of-concept application of the set indicated involvement of ERR (NR3B) and GR (NR3C1) in regulation and resolution of endoplasmic reticulum stress validating suitability of the assembled set for CG. The publicly available NR3 CG library enables translational exploration of NR3 nuclear hormone receptors and investigation of sex differences in phenotypic settings to unveil elusive potential of this important transcription factor family.

## Results

### Identification of CG compound candidates for NR3

**Fig. 2:**
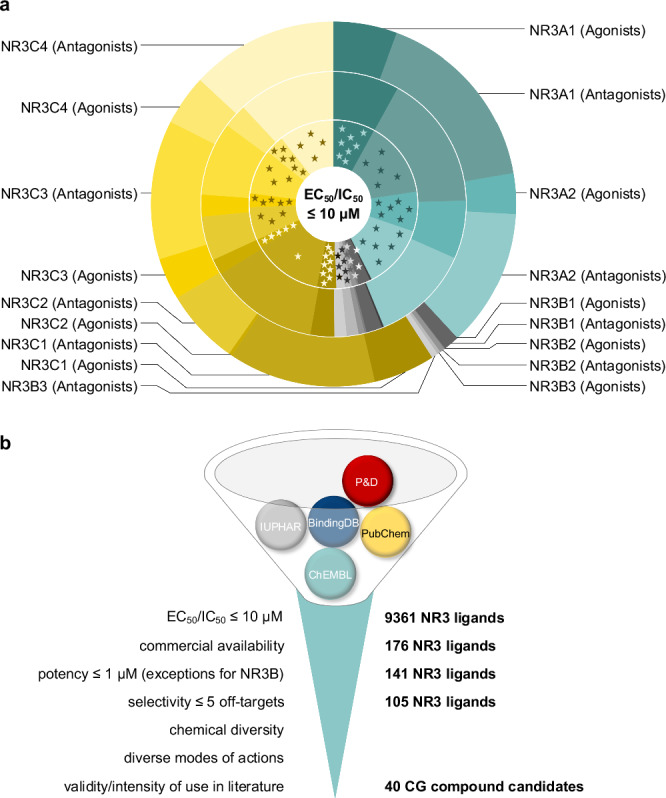
NR3 ligands and selection process. **a** Distribution of the 9361 NR3 agonists and antagonists annotated in public repositories over the individual NR3 receptors (outer circle), the 176 commercially available NR3 ligands (middle circle), and the 141 NR3 ligands complying with the potency cutoff (inner circle). Stars represent the selected 40 CG compound candidates (assigned to NR3 subfamilies considering their respective main targets). Compounds and associated data are listed in Supplementary Table 3. **b** Stepwise filtering process for identification of NR3 ligands as CG compound candidates and numbers of remaining candidates for each step.

9361 NR3 ligands (EC_50_/IC_50_ ≤ 10 µM) are annotated in public compound/bioactivity databases (ChEMBL^28^, PubChem^29^, IUPHAR/BPS^30^, BindingDB^31^, Probes&Drugs^32^; compiled in ref. ^27^) with asymmetric distribution over the nine NR3 receptors (Fig. 2a, Supplementary Data 1), reflecting the varying interest of medicinal chemistry in the different targets. Especially the estrogen receptor-related receptors (ERRα-γ, NR3B1-3) are not well covered by potent and selective ligands.

To identify an optimal set of CG compounds for NR3 receptors, the annotated ligands of the protein family were systematically filtered based on the following criteria (Fig. 2b). To enable a broad use of the selected compounds for CG without restrictions, we prioritized commercially available compounds (Supplementary Table 3). Among them, NR3 ligands with a potency ≤1 µM were considered as candidates with few exceptions for the poorly covered NR3B family, where we also tolerated ≤10 µM potency. Up to five annotated off-targets were accepted in the initial compound selection. Next, we prioritized chemical diversity in the CG set to add orthogonality as chemically different compounds are less likely to share common (unknown) off-targets. Chemical diversity of the compounds was evaluated based on the pairwise Tanimoto similarity computed on Morgan fingerprints^33^ and the candidate combination was optimized for low similarity using a diversity picker. As another level of orthogonality, we included NR3 ligands with diverse modes of action (agonist, antagonist, inverse agonist, modulator, degrader) where available. For NR3 targets with more available ligands complying with these criteria, than desired for the CG set, we eventually considered the validity of the remaining candidates based on the intensity of their use in scientific literature. 40 initial CG compound candidates for NR3 receptors were identified in the filtering process for experimental characterization and validation (Supplementary Table 1) including 15 NR3A ligands, 8 NR3B ligands, and 20 NR3C ligands (considering the respective main target). Of note, some CG compound candidates (e.g., (*Z*)-4-hydroxytamoxifen or diethylstilbestrol) act on more than one NR3 main target.

### Profiling of CG compound candidates

**Fig. 3:**
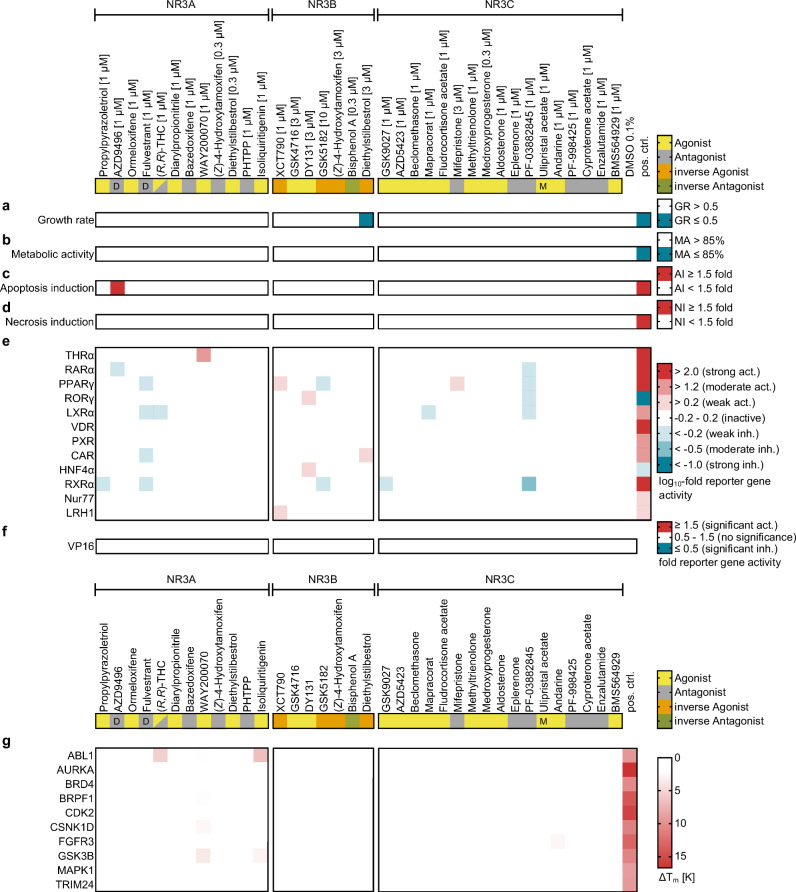
Toxicity and selectivity profiling of the NR3 set. Compounds are labeled according to their mode of action (yellow - agonist, yellow with label M - modulator, grey - antagonist, grey with label D - degrader, orange - inverse agonist, green - inverse antagonist) and grouped by their NR3 subfamily main targets. **a**–**d** Toxicity profiling of NR3 CG compound candidates in HEK293T cells considering growth-rate (**a**), metabolic activity (**b**), apoptosis induction (**c**) and necrosis induction (**d**). Cells were incubated with the test compounds at the indicated concentrations solubilized with 0.1% DMSO for 24 h. Growth-rate was determined based on the change in confluence over 24 h normalized to the change in confluence of cells treated with the 0.1% DMSO control (*n* = 10). Metabolic activity was determined with a water-soluble tetrazolium-8 (WST-8) assay (*n* = 8) after 24 h treatment. Apoptotic cells were detected with a fluorogenic DNA dye coupled to the caspase-3/7 recognition sequence (DEVD) after 24 h treatment and the apoptotic cell count was normalized to 0.1% DMSO treated cells (*n* = 6). Necrotic cells were detected with a red fluorescent dye that labels cells with permeable plasma membranes after 24 h treatment and the necrotic cell count was normalized to 0.1% DMSO treated cells (*n* = 4). **e** NR family selectivity profiling of NR3 CG compound candidates in uniform Gal4-hybrid reporter gene assays for selected NR representing the NR1, NR2, NR4 and NR5 families. Reference ligands are listed in the methods section. The heatmap shows NR mediated activation (red; agonists) and inhibition of reporter gene expression (blue; antagonists and inverse agonists) expressed as mean log_10_ fold reporter activity vs. DMSO ctrl (*n* = 3). **f** Effects of the NR3 CG compound candidates on the ligand-independent transcriptional inducer Gal4-VP16^71,76^ to capture non-specific effects on transcriptional activity. The heatmap shows the mean fold Gal4-VP16 dependent reporter gene activity vs. DMSO ctrl (*n* = 3). **g** Profiling of NR3 CG compound candidates for binding to a panel of highly ligandable liability off-targets that mediate strong phenotypes upon inhibition. Compounds were screened at 20 µM by differential scanning fluorimetry (DSF) with the recombinant proteins (2 µM). Positive controls (pos. ctrl) are listed in the methods section. The heatmap shows the mean ΔT_m_ calculated by Boltzmann fit (*n* = 2).

The selection of 40 CG candidates was acquired from commercial vendors (purity ≥95%) and applied to initial toxicity screening. Cytotoxicity was determined in HEK293T cells considering growth-rate, metabolic activity as well as apoptosis and/or necrosis induction to evaluate suitability of the compounds for CG application in cellular settings (Fig. 3a–d, Supplementary Fig. 1, Supplementary Data 1). The candidates were generally well tolerated at concentrations >>EC_50_/IC_50_ that we recommend for CG application with only four compounds showing mild toxic effects. Diethylstilbestrol reduced the growth-rate at 3 µM but was non-toxic at 0.3 µM. Additionally, AZD9496, ethinylestradiol and budesonide (3 µM) mediated slight apoptosis induction without relevant effects on growth-rate, metabolic activity and necrosis. In terms of cytotoxicity, all candidates thus appeared suitable for inclusion in an NR3 CG set.

To probe selectivity of the candidates in the NR superfamily all compounds were tested for agonistic, antagonistic and inverse agonistic activity in uniform hybrid reporter gene assays^34^ on twelve receptors representing the NR1 (THRα, RARα, PPARγ, RORγ, LXRα, VDR, PXR, CAR), NR2 (HNF4α, RXRα), NR4 (Nur77) and NR5 (LRH1) families (Fig. 3e, Supplementary Fig. 2b, Supplementary Data 1). At concentrations >>EC_50_/IC_50_ for the respective NR3 target, the candidates with exception of biochanin A were favorably selective with few and non-overlapping off-target activities indicating the compounds as suitable for CG.

Next, we screened the candidates for binding to a panel of liability targets by differential scanning fluorimetry (DSF, Fig. 3g, Supplementary Fig. 2c, Supplementary Data 1). This panel comprised ten highly ligandable kinases and bromodomains whose modulation causes strong phenotypes which would compromise use in CG^26^. At 20 µM test concentration, the NR3 CG candidates showed only few and weak interactions with the liability target proteins, and, importantly, the candidate sets for NR3 subfamilies had no common off-targets. The liability screening results thus supported suitability of all candidates for CG, too.

### Assembly and characteristics of the NR3 CG set

**Fig. 4:**
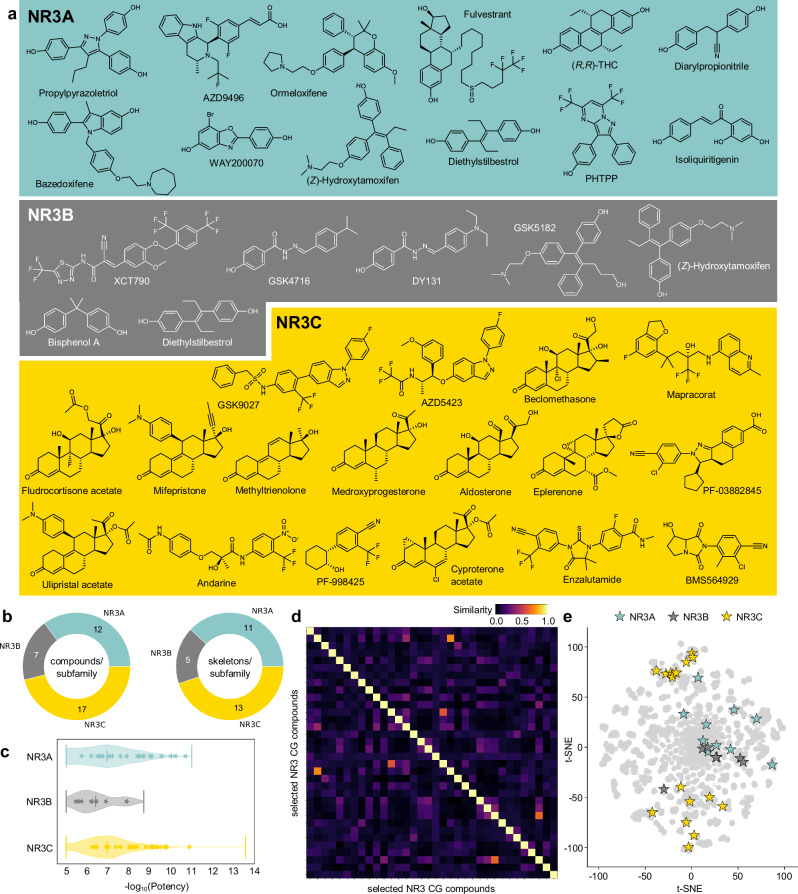
Final set of 34 NR3 CG compounds. **a** Chemical structures and most common names of the selected NR3 CG compounds. **b** Pie charts of the number of compounds per subfamily and the number of diverse skeletons per NR3 subfamily. **c** Potency distribution of known NR3 ligands (≤10 μM from dataset^27^) with selected CG compounds highlighted as stars, shown as the negative decadic logarithm of potency. **d** Heatmap of the pairwise Jaccard-Tanimoto similarity of the selected NR3 CG compounds computed on Morgan fingerprints. Axes represent the selected NR3 CG compounds. **e** Distribution of known NR3 ligands (grey, ≤10 μM from dataset^27^) and the selected NR3 CG compounds (highlighted as stars and colored by NR3 subfamilies) visualized by dimensionality reduction with t-distributed stochastic neighbor embedding (t-SNE) computed on Morgan fingerprints. The axes represent the t-SNE dimensions.

**Fig. 5:**
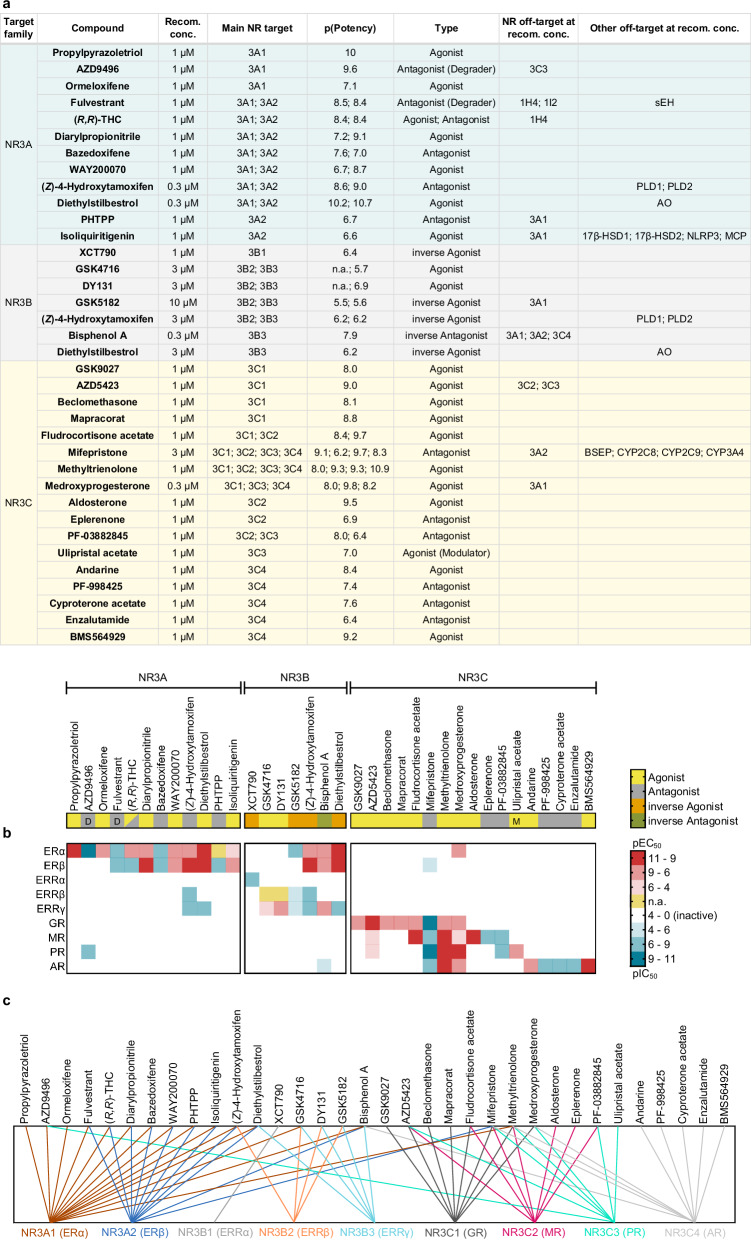
Characteristics of the selected NR3 CG compounds. **a** Recommended concentrations (Recom. conc.) refer to the recommended use of each compound in CG considering potency on the main NR3 target(s), activity type, NR off-targets and known off-targets outside the NR family. p(Potency) values refer to the pEC_50_ for agonists and to pIC_50_ for inverse agonists/antagonists reported in literature (n.a. no data available). Colors denote different NR3 subfamilies and match with the colors in Fig. 4a. Known off-targets of selected NR3 CG compounds are soluble epoxide hydrolase (sEH), phospholipase D1/2 (PLD1/2), aldehyde oxidase (AO), 17β-hydroxysteroid dehydrogenase 1/2 (17β-HSD1/2), nod-like receptor 3 (NLRP3), multi-catalytic protease (MCP), bile salt export pump (BSEP) and several cytochrome P450 (CYP) isoforms. **b** NR3 modulation profiles of the selected CG compounds. Compounds are labeled according to their mode of action (yellow - agonist, yellow with label M - modulator, grey - antagonist, grey with label D - degrader, orange - inverse agonist, green - inverse antagonist) and grouped by their NR3 subfamily main targets. The heatmap shows the potency expressed as pEC_50_ (red; agonists) or pIC_50_ (blue; antagonists and inverse agonists) reported in literature. For a few compounds, only single-point activity data but no EC_50_/IC_50_ have been reported (yellow; n.a.). **c** Correlations between the selected CG compounds and their NR3 targets. Multiple NR3 ligands with diverse activity profiles in the CG set enable deconvolution of phenotypic effects to NR3 receptors.

Based on the favorable results from toxicity and selectivity profiling, we assembled a final set of 34 NR3 CG compounds by rational comparison and selection (Figs. 4 and 5). From the 40 evaluated candidates, we removed the ER (NR3A) degrader ARN810 as this mode-of-action was sufficiently represented by AZD9496 and fulvestrant, which have less off-targets than ARN810 (Supplementary Fig. 2, Supplementary Tables 1, 2, Supplementary Data 1). Similarly, we dropped ethinylestradiol in favor of the more selective and less toxic ER (NR3A) agonists (*R*,*R*)-THC and diarylpropionitrile, the GR/MR (NR3C1/NR3C2) agonist fluticasone propionate in favor of the more potent and selective fludrocortisone acetate, and the NR3C antagonist *Z*-guggulsterone in favor of the more potent and substantially more selective PF-03882845. Additionally, biochanin A and budesonide were removed for their less preferrable selectivity profiles within the NR3 family (Supplementary Fig. 2, Supplementary Table 1, 2, Supplementary Data 1).

The final CG set fully covers the NR3 family with 12 NR3A ligands, 7 NR3B ligands and 17 NR3C ligands (Figs. 4a, b, and 5a, b) and includes at least two modes of action with activating and inhibiting ligands for every NR3 subfamily. As intended, the compound collection exhibited high chemical diversity with low pairwise similarity computed on Morgan fingerprints (Fig. 4d) and high scaffold diversity with the 34 compounds representing 29 different skeletons (Fig. 4b).

All selected NR3A and NR3C ligands exhibit sub-micromolar potency on their targets reported in the literature and are suitable for application in CG at 0.3 µM or 1 µM concentration to ensure a full on-target effect with minimal off-target activities. The poorly explored NR3B family for which substantially less ligands are available required inclusion of less potent CG compounds that have to be used at higher concentrations (3 µM or 10 µM) to achieve a full on-target effect. Recommended concentrations of all selected compounds for application in CG (Fig. 5a, b) have been validated to be non-toxic in cellular setting and modulate the intended NR3 receptor with favorable selectivity within the NR family. Additionally, the combination of several ligands with high chemical diversity in the CG set accounts for unknown off-targets outside the target family which cannot be excluded but likely do not overlap in the chemically diverse ligand collection per target thus enabling deconvolution of phenotypic outcomes observed with the CG library to individual NR3 receptors (Fig. 5c).

### Application of the NR3 CG set

**Fig. 6:**
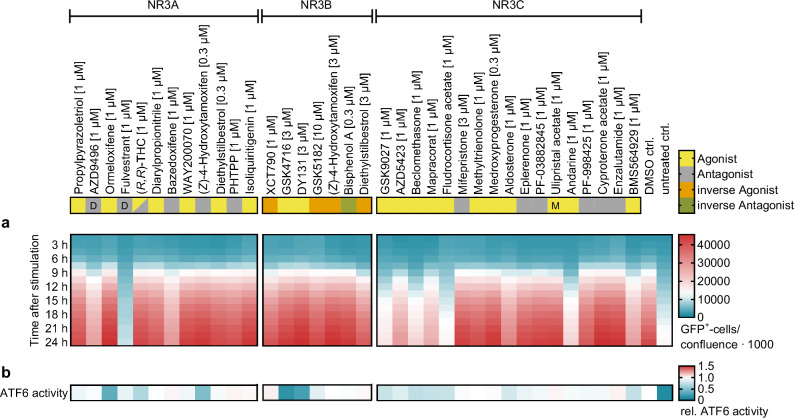
Application of the NR3 CG set. Compounds are labeled according to their mode of action (yellow - agonist, yellow with label M - modulator, grey - antagonist, grey with label D - degrader, orange - inverse agonist, green - inverse antagonist) and grouped by their NR3 subfamily main targets. **a** Effects of the NR3 CG compounds on NF-κB activity in ionomycin/phorbol-12-myristat-13-acetate (PMA) treated HEK293 NF-κB reporter cells. The heatmap shows the mean number of GFP expressing cells normalized to confluence per well; *n* = 3. **b** Effects of the NR3 CG compounds on ATF6 activity in tunicamycin treated Hela ATF6 reporter cells. The heatmap shows the mean relative ATF6 reporter activity normalized to DMSO ctrl; *n* = 5.

For a proof-of-concept application, we probed the impact of the final NR3 CG set on the inflammatory response to ionomycin/ phorbol-12-myristat-13-acetate (PMA) by observing NF-κB activity in HEK293 cells (Fig. 6a, Supplementary Data 1). GR agonists (GSK9027, AZD5423, beclomethasone, mapracorat, fludrocortisone acetate) consistently delayed and strongly diminished NF-κB activity in line with their anti-inflammatory properties^35,36^. Additionally, an anti-inflammatory effect was evident for AR activation (andarine, BMS564929) while AR antagonists (PF-998425, cyproterone acetate, enzalutamide) tended to enhance NF-κB activity. An opposite trend was observable for ER with inhibitors (AZD9496, fulvestrant and bazedoxifene) reducing the inflammatory response and agonists (diarylpropionitrile, propylpyrazoletriol, ormeloxifene, (*R*,*R*)-THC) having no impact on NF-κB activity. These results are in line with previous reports on pro-inflammatory effects of ER activation^37,38^ and AR agonist mediated anti-inflammatory activity^39,40^.

Next, we used the CG set to probe potential NR3-mediated effects in endoplasmic reticulum stress by monitoring activity of the activating transcription factor 6 (ATF6), which is involved in the unfolded protein response and activated in endoplasmic reticulum stress^41–43^. Hela cells treated with tunicamycin to induce endoplasmic reticulum stress displayed markedly enhanced ATF6 activity (Fig. 6b, Supplementary Data 1). ERR agonists (GSK4716, DY131) strongly ameliorated the tunicamycin-induced endoplasmic reticulum stress while the ERR inhibiting CG compounds (XCT790, GSK5182, (*Z*)-4-hydroxytamoxifen (3 µM), diethylstilbestrol) lacked this effect. Consistently weaker ATF6 activity was also evident for GR agonists (GSK9027, AZD5423, beclomethasone, mapracorat, fludrocortisone acetate) but not the antagonist mifepristone. These CG results thus point to an involvement of ERR and GR in endoplasmic reticulum stress regulation and resolution that might be therapeutically explored.

ER modulators, in contrast, displayed inconsistent effects on ATF6 activity. While the ER agonists ormeloxifene, (*R*,*R*)-THC, and (*Z*)-4-hydroxytamoxifen (0.3 µM) mediated resolution of the tunicamycin-induced endoplasmic reticulum stress, the further agonists propylpyrazoletriol, diarylpropionitrile, WAY20070 and diethylstilbestrol failed to reduce ATF6 activity indicating that the effects of ormeloxifene, (*R*,*R*)-THC, and (*Z*)-4-hydroxytamoxifen were not ER-mediated. In line with this interpretation, endoplasmic reticulum stress resolving activity has been reported for estrogen in various cell types but this effect was mainly linked to the membrane estrogen receptor GPER^44–47^.

The proof-of-concept applications thus demonstrated that the NR3 CG set is suitable to correlate phenotypic outcomes with molecular targets and to generate new biological hypotheses. Despite the vast knowledge on NR3 nuclear hormone receptors, it may enable unprecedented discoveries and open new therapeutic opportunities.

## Discussion

Nuclear receptors of the NR3 family act as cellular steroid hormone sensors and translate endocrine signals into transcriptional responses. They operate innumerable processes and have been identified as highly attractive drug targets which is reflected by glucocorticoids as most widely used strong anti-inflammatory drugs or estrogens as female contraceptives. Despite several success stories of NR3 ligands as drugs, the protein family is not comprehensively explored for its therapeutic potential. With increasing availability of highly sophisticated in vitro models for phenotypic studies on many pathologies^48–51^, CG enables systematic exploration of entire protein families for new therapeutic opportunities. For such purposes, we have assembled a CG set covering the NR3 family with carefully selected best-in-class chemical tools.

In CG libraries for target identification and validation, several ligands for one target are combined to account for incomplete selectivity of the utilized compounds^23,26^. Therefore, we have not only considered potency and selectivity as key attributes for chemical tools but also optimized the chemical diversity of the selected compounds to add chemical orthogonality and reduce the probability of common unknown off-targets according to the similarity-property principle^52,53^. Additionally, we focused on commercially available NR3 ligands to enhance accessibility of the set to a broad community.

Extensive annotation of chemical tools is essential for their application especially in CG and, therefore, we have broadly tested the CG compound candidates for cytotoxicity, nuclear receptor selectivity, unspecific effects on transcription and interaction with a panel of liability targets with strong phenotypic impact. A few candidates were excluded based on this profiling and the selected compounds revealed clean profiles supporting their confident use in CG. The resulting collection of 34 CG compounds fully covers the NR3 family as a chemical tool to explore these important transcription factors from a translational perspective.

Preliminary application of this NR3 CG set in functional cellular models provided proof-of-concept for its suitability to expose target-effect relationships: The anti-inflammatory GR agonists^35,36^ expectedly counteracted ionomycin/PMA-induced NF-κB activity in HEK293 cells and our results for NF-κB suppression by ER inhibitors and AR activators support similar observations in previous studies^37–40^. In an ATF6 reporter cell line, application the CG set revealed an impact of several NR3 receptors on endoplasmic reticulum stress. While such activity has been described, e.g., for single ER and GR modulators^54–57^, the consistent effects observed for the GR agonist and ERR modulator subsets provide an additional level of confidence for direct involvement of these receptors in regulating endoplasmic stress responses and the pronounced and uniform impact of ERR agonists on ATF6 activity may be worth further evaluation. These results from functional cellular assays showcase the potential of the curated NR3 modulator set to link NR3 receptors to phenotypic effects and encourage further application of the CG approach to this protein family. Phenotypic in vitro models are on the rise and can mimic various tissues and associated pathologies ranging from cancer over infectious diseases and metabolic dysfunction to neurodegeneration^48–51^. Allowing substantially higher throughput than traditional preclinical models, such 3D cellular systems are a perfect match with the concept of CG to explore new target-effect relationships. Application of the NR3 CG set according to this concept in such modern in vitro models may unveil new therapeutic opportunities.

## Methods

### CG compound selection

#### Computational methods for CG candidate compound selection

CG compound candidate selection was performed with Konstanz Information Miner (KNIME, version 4.5)^58^ software with RDKit (version 2022.09.1) nodes. Compounds were extracted from a recently compiled dataset^27^ containing multiple annotated bioactivities from five public databases (PubChem^29^, ChEMBL^28^, IUPHAR/BPS^30^, BindingDB^31^ and Probes&Drugs^32^). The compounds were prefiltered for bioactivity ≤1 µM (NR3A, NR3C) and ≤10 µM (NR3B) on the intended main target based on the published bioactivities. Commercial availability was determined by combining vendor databases (Tocris, Sigma, Cayman Chemicals, Selleckchem and MedChemExpress) with the compound selection using ChEMBL IDs and SMILES. The number of off-targets was determined by searching the dataset for other targets of the preselected compounds with annotated bioactivities at ≤10 µM. Chemical diversity of the CG candidate compounds was evaluated based on Morgan fingerprints^33^ calculated using the RDKit Fingerprint node with following settings: Fingerprint type = Morgan, number of bits =1024 and radius =2. For skeleton analysis, RDKit Find Murcko Scaffolds with the setting create frameworks was used. Up to 10 chemically diverse molecules and skeletons were selected for each target using the RDKit Diversity Picker. Bioactivity values annotated in the database were compared/validated with the original literature and the indicated mode of action before candidates were selected for profiling. Chemical property analysis was done using KNIME and python (version 3.8)^59^. The similarity heatmap was generated using Tanimoto similarity^60^ computed on Morgan fingerprints. For the t-SNE and potency analysis, all NR3 compounds with annotated bioactivity ≤10 µM were selected from the dataset^27^, and the t-SNE was computed based on Morgan fingerprints (from RDKit in KNIME) using scikit-learn (version 1.3.)^61^ with the following settings: n_components = 2, perplexity = 30, learning_rate = auto, init = pca.

### Biological quality control

#### Evaluation of cytotoxicity

HEK293T cells (German Collection of Microorganisms and Cell Culture GmbH, DSMZ; #ACC305) were cultured in DMEM high glucose, supplemented with 10% fetal calf serum (FCS), sodium pyruvate (1 mM), penicillin (100 µ/mL) and streptomycin (100 µg/mL) at 37 °C and 5% (v/v) CO_2_, and seeded in pre-coated (10 µg/mL collagen G solution (Merck KgaA; #L7213) for 30 min at 37 °C) 96-well plates (2 × 10^5^ cells per well). After 24 h, the cells were treated with the respective NR3 CG compound candidate at the recommended concentration, bexarotene (100 µM) or doxorubicin (30 µM) as positive controls in DMEM high glucose supplemented with 0.1% FCS, penicillin (100 µ/mL), streptomycin (100 µg/mL) and 0.1% DMSO, or 0.1% DMSO alone as an untreated control. Each sample was prepared in at least four biologically independent replicates. After 24 h incubation, the medium was aspirated, and the cells were incubated for 30 min with staining medium containing 1 µM NucView® 405 fluorogenic caspase-3 substrate (Biotium, Fremont, California, USA; #10405) and Live-or-Dye Nuc-Fix Red (0.05x, Biotium, Fremont, California, USA; #32010-T) to detect apoptosis and necrosis, respectively. After incubation, a total of six fluorescence images per well at 10x magnification were taken to detect NucView®-positive (Ex: 381–400 nm, Em: 414–450 nm) and Live-or-Dye-positive cells (Ex: 543−566 nm, Em: 580–611 nm) using on a Tecan Spark Cyto (Tecan Group AG, Männedorf, Switzerland). Reference readings for background correction and detection of auto-fluorescence were taken at 414–450 nm prior to staining. Thereafter, the medium was aspirated and the cells were incubated for 3 h with 90 µL DMEM high glucose supplemented with 0.2% FCS, penicillin (100 µ/mL) and streptomycin (100 µg/mL) and additionally containing 10 µL Cell Counting Kit 8 solution (CCK-8; MedChemExpress, Monmouth Junction, New York, USA; #HY-K0301), and absorbance was measured at 1 h, 2 h and 3 h of incubation at 450 nm on a Tecan Spark Cyto to assess metabolic activity of the cells. Before drug administration, after the first medium exchange, 24 h after drug administration, and after fluorescence imaging cell confluence was assessed using the Tecan Spark Cyto, to account for changes in cell confluence due to drug administration and cell handling. Compound effects on cell confluence, apoptosis, metabolic activity and necrosis were normalized to the vehicle control of each biological replicate individually.

#### Liability panel screening by DSF

In-house produced recombinantly expressed proteins^62^ of the liability panel targets were diluted in a buffer containing 10 mM HEPES (pH 7.5) and 500 mM NaCl to a concentration of 2 µM. SYPRO Orange (Invitrogen, Carlsbad, California, USA; #S6650) as a fluorescence probe was added at a 1:1000 dilution to the proteins. The protein-dye mixtures were transferred to a 384-well plate with 20 µL volume, and the NR3 CG compound candidates were added at a final concentration of 20 µM using a Echo 525 liquid handler (Labcyte, San Jose, California, USA; #001-10080). Temperature-dependent unfolding profiles were measured on a QuantStudio™ 5 real-time PCR machine (Thermo Fisher Scientific, Waltham, Massachusetts, USA; #A28140). Excitation and emission filters were set to 465 nm and 590 nm, respectively. The temperature was increased 3 °C per minute to a maximum temperature of 85 °C. Experiments were run as technical duplicates. As positive controls (pos. ctrl.) staurosporine (ABL1, AURKA, CDK2, FGFR3 and GSK3B), (+)-JQ1 (BRD4), GSK6853 (BRPF1), PK016714a (CSNK1D), GDC-0994 (MAPK1) and IACS-9571 (TRIM24) were used with 20 µM each. Data were analyzed with the internal Thermal Shift Software (version 1.4, Thermo Fisher Scientific) using the Boltzmann equitation to determine the inflection point of the transition curve. Differences in melting temperature are given as ∆Tm in K.

### CG compound profiling

#### Gal4 hybrid reporter gene assays

HEK293T cells (ATCC, Manassas, Virgina, USA; #CRL-3216) were cultured to a maximal confluence of 70–80% in DMEM, high glucose, supplemented with 10% FCS, sodium pyruvate (1 mM), penicillin (100 U/mL), and streptomycin (100 μg/mL) at 37 °C and 5% CO_2_ (v/v), and seeded in clear 96-well plates (4 × 10^4^ cells per well). After 20–24 h, medium was changed to Opti-MEM without supplements and the cells were transiently transfected using Lipofectamine™ LTX/Plus™ Reagents (Invitrogen, Carlsbad, California, USA; #15338100) according to the manufacturer’s protocol with pFR-Luc (reporter gene; Stratagene, Agilent Technologies, Santa Clara, California, USA; #219050), pRL-SV40 (control gene for normalization of transfection efficiency and cell growth; Promega, Fitchburg, Wisconsin, USA; #E2231) and one pFA-CMV-hNR-LBD clone coding for the hinge region and ligand binding domain (LBD) of the human NR of interest. Five hours after transfection, the cells were incubated with the NR3 CG compound candidates at the recommended concentrations by changing the medium to Opti-MEM supplemented with penicillin (100 µ/mL) and streptomycin (100 µg/mL) additionally containing 0.1% DMSO and the respective test compound or 0.1% DMSO alone as untreated control. Firefly (reporter) and renilla (control) luminescence were measured after 14–16 h incubation using the Dual-Glo® Luciferase Assay System (Promega, Fitchburg, Wisconsin, USA; #E2980) according to manufacturer’s protocol on a Tecan Spark® 10M multimode microplate reader (Tecan Group AG, Männedorf, Switzerland). To obtain relative light units (RLU) firefly luminescence was divided by renilla luminescence and multiplied by 1000. Fold activation was obtained by dividing the mean RLU of a test sample by the mean RLU of the DMSO (0.1%) control. Experiments were performed in singlets in at least three biologically independent repeats. The following pFA-CMV-hNR-LBD clones and reference ligands were used: THRα (pFA-CMV-hTHRα-LBD^63^, 1 µM T3), RARα (pFA-CMV-hRARα-LBD^64^, 1 µM retinoic acid), PPARγ (pFA-CMV-hPPARγ-LBD^65^, 1 µM rosiglitazone), RORγ (pFA-CMV-hRORγ-LBD^66^, 1 µM SR1001), LXRα (pFA-CMV-hLXRα-LBD^67^, 1 µM T0901317), VDR (pFA-CMV-hVDR-LBD^64^, 1 µM calcitriol), PXR (pFA-CMV-hPXR-LBD^64^, 1 µM SR12813), CAR (pFA-CMV-hCAR-LBD^64^, 1 µM CITCO), HNF4α (pFA-CMV-hHNF4α-LBD^68^, 30 µM compound 9 from ref. ^68^), RXRα (pFA-CMV-hRXRα-LBD^64^, 1 µM bexarotene), Nur77 (pFA-CMV-hNur77-LBD^69^, 100 µM amodiaquine), and LRH1 (pFA-CMV-hSF1-LBD^70^, in-house compound). pECE-SV40-Gal4-VP16^71^ (gift from Lea Sistonen; Addgene, Watertown, Massachusetts, USA; #71728) encoding the ligand-independent transcriptional inducer Gal4-VP16 was used instead of a pFA-CMV-hNR-LBD clone to test for unspecific compound effects on reporter activity.

### Assays for NR3 CG set application

#### NF-kB activity assay

HEK293 cells containing a GFP reporter gene for NF-kB activity (NF-κB Leeporter™ GFP Reporter-HEK293 Cell Line, Abeomics Inc., San Diego, California, USA; #14-700ACL) were cultured in DMEM supplemented with 10% heat-inactivated FCS, penicillin (100 μ/mL), streptomycin (100 μg/mL) and puromycin (3 µg/ml) at 37 °C and 5% (v/v) CO_2_ and seeded in 96-well plates (3 × 10^4^ cells per well). After 24 h, cells were incubated with the test compounds solubilized with 0.1% DMSO in the same medium without puromycin. After 4 h incubation, ionomycin (100 ng/mL) and phorbol-12-myristat-13-acetate (PMA, 10 ng/mL) was added, and the cells were imaged for confluence and GFP expression every 90 min. over 24 h using a Tecan SPARK Cyto (Tecan). The number of GFP expressing cells was normalized to confluence in each sample. Each sample was repeated in three biologically independent experiments.

#### Endoplasmic reticulum stress assay

Hela cells containing a Renilla luciferase reporter gene for ATF6 activity (ATF6 Leeporter™ Luciferase Reporter-HeLa Cell Line, Abeomics Inc.; #14-138ACL) were cultured in DMEM supplemented with 10% heat-inactivated FCS, penicillin (100 μ/mL), streptomycin (100 μg/mL) and puromycin (3 µg/ml) at 37 °C and 5% (v/v) CO_2_ and seeded in 96-well plates (1 × 10^4^ cells per well). After 24 h, cells were incubated with the test compounds solubilized with 0.1% DMSO in the same medium without puromycin. After 4 h incubation, tunicamycin (100 ng/mL) was added. After another 18 h incubation, cells were assayed for renilla luminescence using DualGlo reagent (Promega) on a Tecan SPARK Cyto (Tecan). Test samples were normalized to the 0.1% DMSO control. Each sample was repeated in five biologically independent experiments.

### Reporting summary

Further information on research design is available in the Nature Portfolio Reporting Summary linked to this article.

## Supplementary information

Transparent Peer Review file

Supplementary Information

Description of Additional Supplementary Files

Supplementary Data 1

reporting summary

## Data Availability

All data supporting the results of this study are available in the Supplementary Information. Source data are provided with this paper in Supplementary Data 1.

## References

[1] Cain, D. W. & Cidlowski, J. A. Immune regulation by glucocorticoids. *Nat. Rev. Immunol.* **17**, 233–247 (2017). 28192415 10.1038/nri.2017.1 PMC9761406

[2] Vandewalle, J., Luypaert, A., De Bosscher, K. & Libert, C. Therapeutic mechanisms of glucocorticoids. *Trends Endocrinol. Metab.* **29**, 42–54 (2018). 29162310 10.1016/j.tem.2017.10.010

[3] Briet, M. & Schiffrin, E. L. Aldosterone: effects on the kidney and cardiovascular system. *Nat. Rev. Nephrol.* **6**, 261–273 (2010). 20234356 10.1038/nrneph.2010.30

[4] Pillerová, M. et al. On the role of sex steroids in biological functions by classical and non-classical pathways. An update. *Front. Neuroendocrinol.* **62**, 100926 (2021). 34089761 10.1016/j.yfrne.2021.100926 PMC8523217

[5] Lösel, R. & Wehling, M. Nongenomic actions of steroid hormones. *Nat. Rev. Mol. Cell Biol.* **4**, 46–56 (2003). 12511868 10.1038/nrm1009

[6] Norman, A. W., Mizwicki, M. T. & Norman, D. P. G. Steroid-hormone rapid actions, membrane receptors and a conformational ensemble model. *Nat. Rev. Drug Discov.* **3**, 27–41 (2004). 14708019 10.1038/nrd1283

[7] Lu, N. Z. et al. International Union of Pharmacology. LXV. The pharmacology and classification of the nuclear receptor superfamily: glucocorticoid, mineralocorticoid, progesterone, and androgen receptors. *Pharm. Rev.* **58**, 782–797 (2006). 17132855 10.1124/pr.58.4.9

[8] Dahlman-Wright, K. et al. International Union of Pharmacology. LXIV. Estrogen receptors. *Pharm. Rev.* **58**, 773–781 (2006). 17132854 10.1124/pr.58.4.8

[9] Isigkeit, L. & Merk, D. Opportunities and challenges in targeting orphan nuclear receptors. *Chem. Commun.* **59**, 4551–4561 (2023). 10.1039/d3cc00954h 37000699

[10] De Bosscher, K., Desmet, S. J., Clarisse, D., Estébanez-Perpiña, E. & Brunsveld, L. Nuclear receptor crosstalk — defining the mechanisms for therapeutic innovation. *Nat. Rev. Endocrinol.* **16**, 363–377 (2020). 32303708 10.1038/s41574-020-0349-5

[11] Rhen, T. & Cidlowski, J. A. Antiinflammatory action of glucocorticoids — new mechanisms for old drugs. *N. Engl. J. Med.* **353**, 1711–1723 (2005). 16236742 10.1056/NEJMra050541

[12] Teal, S. & Edelman, A. Contraception selection, effectiveness, and adverse effects: a review. *J. Am. Med. Assoc.* **326**, 2507–2518 (2021). 10.1001/jama.2021.21392 34962522

[13] Patel, H. K. & Bihani, T. Selective estrogen receptor modulators (SERMs) and selective estrogen receptor degraders (SERDs) in cancer treatment. *Pharm. Ther.* **186**, 1–24 (2018). 10.1016/j.pharmthera.2017.12.012 29289555

[14] Risbridger, G. P., Davis, I. D., Birrell, S. N. & Tilley, W. D. Breast and prostate cancer_more similar than different. *Nat. Rev. Cancer* **10**, 205–212 (2010). 20147902 10.1038/nrc2795

[15] Kintscher, U., Bakris, G. L. & Kolkhof, P. Novel non-steroidal mineralocorticoid receptor antagonists in cardiorenal disease. *Br. J. Pharm.* **179**, 3220–3234 (2022). 10.1111/bph.15747 34811750

[16] Cutolo, M. & Straub, R. H. Sex steroids and autoimmune rheumatic diseases: state of the art. *Nat. Rev. Rheumatol.* **16**, 628–644 (2020). 33009519 10.1038/s41584-020-0503-4

[17] Forsyth, K. S., Jiwrajka, N., Lovell, C. D., Toothacre, N. E. & Anguera, M. C. The conneXion between sex and immune responses. *Nat. Rev. Immunol.* **24**, 487–502 (2024). 38383754 10.1038/s41577-024-00996-9 PMC11216897

[18] Mauvais-Jarvis, F. Sex differences in energy metabolism: natural selection, mechanisms and consequences. *Nat. Rev. Nephrol.* **20**, 56–69 (2024). 37923858 10.1038/s41581-023-00781-2

[19] Antwi, M. B. et al. Unlocking therapeutic potential: exploring cross-talk among emerging nuclear receptors to combat metabolic dysfunction in steatotic liver disease. *NPJ Metab. Health Dis.* **2**, 13 (2024). 10.1038/s44324-024-00013-6 PMC12118758 40603501

[20] Jacob, S. M. et al. Brain–body mechanisms contribute to sexual dimorphism in amyotrophic lateral sclerosis. *Nat. Rev. Neurol.* **20**, 475–494 (2024). 38965379 10.1038/s41582-024-00991-7

[21] Arevalo, M. A., Azcoitia, I. & Garcia-Segura, L. M. The neuroprotective actions of oestradiol and oestrogen receptors. *Nat. Rev. Neurosci.* **16**, 17–29 (2015). 25423896 10.1038/nrn3856

[22] Bredel, M. & Jacoby, E. Chemogenomics: an emerging strategy for rapid target and drug discovery. *Nat. Rev. Genet.* **5**, 262–275 (2004). 15131650 10.1038/nrg1317

[23] Chaikuad, A. & Merk, D. An Introduction to chemogenomics. in *Methods in Molecular Biology* (eds Chaikuad, A. & Merk, D.) 2706 1–10 (Humana, New York, 2023). 10.1007/978-1-0716-3397-7_1 37558937

[24] Jones, L. H. & Bunnage, M. E. Applications of chemogenomic library screening in drug discovery. *Nat. Rev. Drug Discov.* **16**, 285–296 (2017). 28104905 10.1038/nrd.2016.244

[25] Wells, C. I. et al. The Kinase Chemogenomic Set (KCGS): an open scienceresource for kinase vulnerability identification. *Int. J. Mol. Sci.* **22**, 1–18 (2021). 10.3390/ijms22020566 PMC7826789 33429995

[26] Isigkeit, L. et al. Chemogenomics for NR1 nuclear hormone receptors. *Nat. Commun.* **15**, 5201 (2024). 38890295 10.1038/s41467-024-49493-6 PMC11189487

[27] Isigkeit, L., Chaikuad, A. & Merk, D. A consensus compound/bioactivity dataset for data-driven drug design and chemogenomics. *Molecules* **27**, 2513 (2022). 35458710 10.3390/molecules27082513 PMC9028877

[28] Mendez, D. et al. ChEMBL: towards direct deposition of bioassay data. *Nucleic Acids Res.* **47**, D930–D940 (2019). 30398643 10.1093/nar/gky1075 PMC6323927

[29] Kim, S. et al. PubChem in 2021: New data content and improved web interfaces. *Nucleic Acids Res.* **49**, D1388–D1395 (2021). 33151290 10.1093/nar/gkaa971 PMC7778930

[30] Harding, S. D. et al. The IUPHAR/BPS guide to PHARMACOLOGY in 2022: curating pharmacology for COVID-19, malaria and antibacterials. *Nucleic Acids Res.* **50**, D1282–D1294 (2022). 34718737 10.1093/nar/gkab1010 PMC8689838

[31] Gilson, M. K. et al. BindingDB in 2015: A public database for medicinal chemistry, computational chemistry and systems pharmacology. *Nucleic Acids Res.* **44**, D1045–D1053 (2016). 26481362 10.1093/nar/gkv1072 PMC4702793

[32] Skuta, C. et al. Probes & Drugs portal: an interactive, open data resource for chemical biology. *Nat. Methods* **14**, 759–760 (2017). 28753599 10.1038/nmeth.4365

[33] Morgan, H. L. The generation of a unique machine description for chemical structures — a technique developed at chemical abstracts service. *J. Chem. Doc.* **4**, 107–113 (1964).

[34] Heering, J. & Merk, D. Hybrid Reporter Gene Assays: Versatile In Vitro Tools to Characterize Nuclear Receptor Modulators. in *Methods in Molecular Biology* (ed. Badr, M. Z.) 1966, 175–192 (Humana, New York, 2019). 10.1007/978-1-4939-9195-2_14 31041747

[35] Unlap, T. & Jope, R. S. Inhibition of NFkB DNA binding activity by glucocorticoids in rat brain. *Neurosci. Lett.* **198**, 41–44 (1995). 8570092 10.1016/0304-3940(95)11963-w

[36] Barnes, P. J. Anti-inflammatory actions of glucocorticoids: molecular mechanisms. *Clin. Sci.* **94**, 557–572 (1998). 10.1042/cs0940557 9854452

[37] Wu, H. et al. Estrogen acts through estrogen receptor-β to promote mannan-induced psoriasis-like skin inflammation. *Front. Immunol.* **13**, 818173 (2022). 35663991 10.3389/fimmu.2022.818173 PMC9160234

[38] Straub, R. H. The complex role of estrogens in inflammation. *Endocr. Rev.* **28**, 521–574 (2007). 17640948 10.1210/er.2007-0001

[39] Zhang, X. et al. Androgen signaling contributes to sex differences in cancer by inhibiting NF-kB activation in T cells and suppressing antitumor immunity. *Cancer Res.* **83**, 906–921 (2023). 36634207 10.1158/0008-5472.CAN-22-2405

[40] Bupp, M. R. G. & Jorgensen, T. N. Androgen-induced immunosuppression. *Front. Immunol.* **9**, 794 (2018). 29755457 10.3389/fimmu.2018.00794 PMC5932344

[41] Li, M. et al. ATF6 as a transcription activator of the endoplasmic reticulum stress element: thapsigargin stress-induced changes and synergistic interactions with NF-Y and YY1. *Mol. Cell Biol.* **20**, 5096–5106 (2000). 10866666 10.1128/mcb.20.14.5096-5106.2000 PMC85959

[42] Chen, X., Shen, J. & Prywes, R. The Luminal Domain of ATF6 Senses Endoplasmic Reticulum (ER)stress and causes translocation of ATF6 from the ER to the golgi. *J. Biol. Chem.* **277**, 13045–13052 (2002). 11821395 10.1074/jbc.M110636200

[43] Okada, T., Yoshida, H., Akazawa, R., Negishi, M. & Mori, K. Distinct roles of activating transcription factor 6 (ATF6) and double-stranded RNA-activated protein kinase-like endoplasmic reticulum kinase (PERK) in transcription during the mammalian unfolded protein response. *Biochem. J.* **366**, 585–594 (2002). 12014989 10.1042/BJ20020391 PMC1222788

[44] Su, B. et al. Icariin attenuation of diabetic kidney disease through inhibition of endoplasmic reticulum stress via G protein-coupled estrogen receptors. *J. Biomed. Nanotechnol.* **18**, 488–497 (2022). 35484747 10.1166/jbn.2022.3242

[45] Han, Z. W. et al. GPER agonist G1 suppresses neuronal apoptosis mediated by endoplasmic reticulum stress after cerebral ischemia/reperfusion injury. *Neural Regen. Res.* **14**, 1221–1229 (2019). 30804253 10.4103/1673-5374.251571 PMC6425826

[46] Li, R., Wang, Y., Chen, P., Meng, J. & Zhang, H. Inhibiting endoplasmic reticulum stress by activation of G-protein-coupled estrogen receptor to protect retinal astrocytes under hyperoxia. *J. Biochem. Mol. Toxicol.* **35**, e22641 (2021). 32996202 10.1002/jbt.22641

[47] Li, R., Wang, Y., Chen, P., Meng, J. & Zhang, H. G-protein-coupled estrogen receptor protects retinal ganglion cells via inhibiting endoplasmic reticulum stress under hyperoxia. *J. Cell Physiol.* **236**, 3780–3788 (2021). 33151568 10.1002/jcp.30149

[48] Neufeld, L., Yeini, E., Pozzi, S. & Satchi-Fainaro, R. 3D bioprinted cancer models: from basic biology to drug development. *Nat. Rev. Cancer* **22**, 679–692 (2022). 36280768 10.1038/s41568-022-00514-w

[49] Barrila, J. et al. Organotypic 3D cell culture models: Using the rotating wall vessel to study host-pathogen interactions. *Nat. Rev. Microbiol* **8**, 791–801 (2010). 20948552 10.1038/nrmicro2423

[50] Zhou, Z. et al. Harnessing 3D in vitro systems to model immune responses to solid tumours: a step towards improving and creating personalized immunotherapies. *Nat. Rev. Immunol.* **24**, 18–32 (2024). 37402992 10.1038/s41577-023-00896-4

[51] Ko, J. et al. Microfluidic high-throughput 3D cell culture. *Nat. Rev. Bioeng.* **2**, 453–469 (2024).

[52] O’Boyle, N. M. & Sayle, R. A. Comparing structural fingerprints using a literature-based similarity benchmark. *J. Cheminform* **8**, 1–14 (2016). 27382417 10.1186/s13321-016-0148-0 PMC4932683

[53] Johnson, M. A. & Maggiora, G. M. *Concepts and Applications of Molecular Similarity* (Wiley Interscience Publication, New York, 1990).

[54] Das, I. et al. Glucocorticoids alleviate intestinal ER stress by enhancing protein folding and degradation of misfolded proteins. *J. Exp. Med.* **210**, 1201–1216 (2013). 23650437 10.1084/jem.20121268 PMC3674691

[55] Yan, M. et al. Knockdown of NR3C1 inhibits the proliferation and migration of clear cell renal cell carcinoma through activating endoplasmic reticulum stress–mitophagy. *J. Transl. Med.* **21**, 701 (2023). 37807060 10.1186/s12967-023-04560-2 PMC10560440

[56] Kooptiwut, S. et al. Estrogen reduces endoplasmic reticulum stress to protect against glucotoxicity induced-pancreatic β-cell death. *J. Steroid Biochem. Mol. Biol.* **139**, 25–32 (2014). 24120916 10.1016/j.jsbmb.2013.09.018

[57] Su, Q. et al. Inhibition of endoplasmic reticulum stress apoptosis by estrogen protects human umbilical vein endothelial cells through the PI3 Kinase–Akt signaling pathway. *J. Cell Biochem.* **118**, 4568–4574 (2017). 28485890 10.1002/jcb.26120

[58] Berthold, M. R. et al. KNIME: The Konstanz Information Miner. in *Data Analysis, Machine Learning and Applications. Studies in Classification, Data Analysis, and Knowledge* Organiz*at*io*n* (eds Preisach, C., Burkhardt, H., Schmidt-Thieme, L. & Decker, R.) 319–326 (Springer, Berlin, Heidelberg, 2008).

[59] Van Rossum, G. & Drake, F. L. *Python 3 Reference Manual*. (CreateSpace, Scotts Valley, CA, 2009).

[60] Todeschini, R., Ballabio, D. & Consonni, V. Distances and Similarity Measures in Chemometrics and Chemoinformatics. in *Encyclopedia of Analytical Chemistry* 1–40 (Wiley, Hoboken, 2020).

[61] Pedregosa, F. et al. Scikit-learn: machine learning in python. *J. Mach. Learn Res.* **12**, 2825–2830 (2011).

[62] Rak, M. et al. Development of selective Pyrido[2,3-d]pyrimidin-7(8H)-one-based mammalian STE20-Like (MST3/4) kinase inhibitors. *J. Med. Chem.* **67**, 3813–3842 (2024). 38422480 10.1021/acs.jmedchem.3c02217

[63] Gellrich, L. et al. L-thyroxin and the nonclassical thyroid hormone TETRAC are potent activators of PPARÎ. *J. Med. Chem.* **63**, 6727–6740 (2020). 32356658 10.1021/acs.jmedchem.9b02150

[64] Flesch, D. et al. Nonacidic farnesoid X receptor modulators. *J. Med. Chem.* **60**, 7199–7205 (2017). 28749691 10.1021/acs.jmedchem.7b00903

[65] Rau, O. et al. Carnosic acid and carnosol, phenolic diterpene compounds of the labiate herbs rosemary and sage, are activators of the human peroxisome proliferator-activated receptor gamma. *Planta Med.* **72**, 881–887 (2006). 16858665 10.1055/s-2006-946680

[66] Moret, M., Helmstädter, M., Grisoni, F., Schneider, G. & Merk, D. De novo design beam search for automated design and scoring of NovelR OR ligands with machine intelligence**. *Angew Chem. Int. Ed.* **60**, 19477–19482 (2021). 10.1002/anie.202104405 PMC8457062 34165856

[67] Lamers, C. et al. SAR studies on FXR modulators led to the discovery of the first combined FXR antagonistic/TGR5 agonistic compound. *Future Med. Chem.* **8**, 133–148 (2016). 26824277 10.4155/fmc.15.178

[68] Meijer, I. et al. Chemical starting matter for HNF4α ligand discovery and chemogenomics. *Int. J. Mol. Sci.* **21**, 1–11 (2020). 10.3390/ijms21217895 PMC7660650 33114319

[69] Willems, S. et al. Nurr1 modulation mediates neuroprotective effects of statins. *Adv. Sci.* **9**, e2104640 (2022). 10.1002/advs.202104640 PMC9218776 35488520

[70] Vietor, J. et al. Development of a potent Nurr1 agonist tool for in vivo applications. *J. Med. Chem.* **66**, 6391–6402 (2023). 37127285 10.1021/acs.jmedchem.3c00415 PMC10184128

[71] Budzyński, M. A., Puustinen, M. C., Joutsen, J. & Sistonen, L. Uncoupling stress-inducible phosphorylation of heat shock factor 1 from its activation. *Mol. Cell Biol.* **35**, 2530–2540 (2015). 25963659 10.1128/MCB.00816-14 PMC4475925

[72] King, W. J. & Greene, G. L. Monoclonal antibodies localize oestrogen receptor in the nuclei of target cells. *Nature* **307**, 745–747 (1984). 6700704 10.1038/307745a0

[73] Picard, D., Kumart, V., Chambont, P. & Yamamoto, K. R. Signal transduction by steroid hormones: nuclear localization is differentially regulated in estrogen and glucocorticoid receptors. *Cell Regul.* **1**, 291–299 (1990). 2100202 10.1091/mbc.1.3.291 PMC361473

[74] Htun, H., Holth, L. T., Walker, D., Davie, J. R. & Hager, G. L. Direct visualization of the human estrogen receptor α reveals a role for ligand in the nuclear distribution of the receptor. *Mol. Biol. Cell* **10**, 471–486 (1999). 9950689 10.1091/mbc.10.2.471 PMC25181

[75] Saha, S., Dey, S. & Nath, S. Steroid hormone receptors: links with cell cycle machinery and breast cancer progression. *Front. Oncol.* **11**, 1–11 (2021). 10.3389/fonc.2021.620214 PMC7994514 33777765

[76] Sadowski, I., Ma, J., Triezenbergt, S. & Ptashne, M. GAL4-VP16 is an unusually potent transcriptional activator. *Nature* **335**, 563–564 (1988). 3047590 10.1038/335563a0

