# Supplementary material for: Chemogenomics for steroid hormone receptors (NR3)
Source: Commun Chem. 2025 Feb 3;8:29. doi: 10.1038/s42004-025-01427-z (PMC11790914; doi:10.1038/s42004-025-01427-z)
Supplement: Supplementary file 2 — Supplementary Information [file 42004_2025_1427_MOESM2_ESM.pdf]

Chemogenomics for steroid hormone receptors (NR3)

Espen Schallmayer[1], Laura Isigkeit[1], Lewis Elson[1], Susanne Müller[1], Stefan Knapp[1], Julian A. Marschner[2], Daniel Merk[1,2]*

[1] Goethe Universität Frankfurt, Institute of Pharmaceutical Chemistry, 60438 Frankfurt, Germany
[2] Ludwig-Maximilians-Universität München, Department of Pharmacy, 81377 Munich, Germany
* daniel.merk@cup.lmu.de

Table of Contents

Supplementary Figures and Tables 2
Supplementary References 17

Supplementary Figures

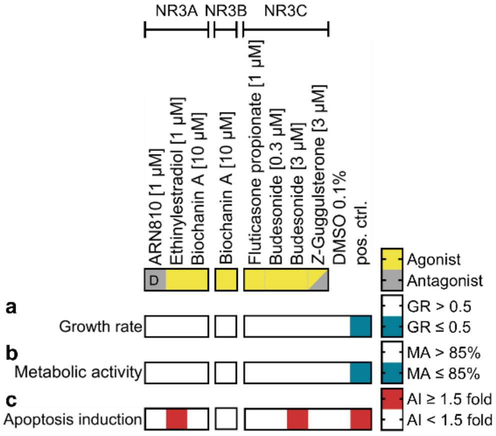

Supplementary Figure 1. Toxicity profiling of the excluded NR3 CG compound candidates. Compounds are labeled according to their mode of action (yellow - agonist, grey - antagonist, grey with label D - degrader) and grouped by their NR3 subfamily main targets. HEK293T cells were incubated with the test compounds at the indicated concentrations solubilized with 0.1% DMSO for 24 h. **a** Growth-rate (GR) was determined based on the change in confluence over 24 h normalized to the change in confluence of cells treated with the 0.1% DMSO control (n=6). **b** Metabolic activity (MA) was determined with a water-soluble tetrazolium-8 (WST-8) assay after 24 h treatment (n=6). **c** Apoptosis induction (AI) was detected with a fluorogenic DNA dye coupled to the caspase-3/7 recognition sequence (DEVD) after 24 h treatment and the apoptotic cell count was normalized to 0.1% DMSO treated cells (n=6).

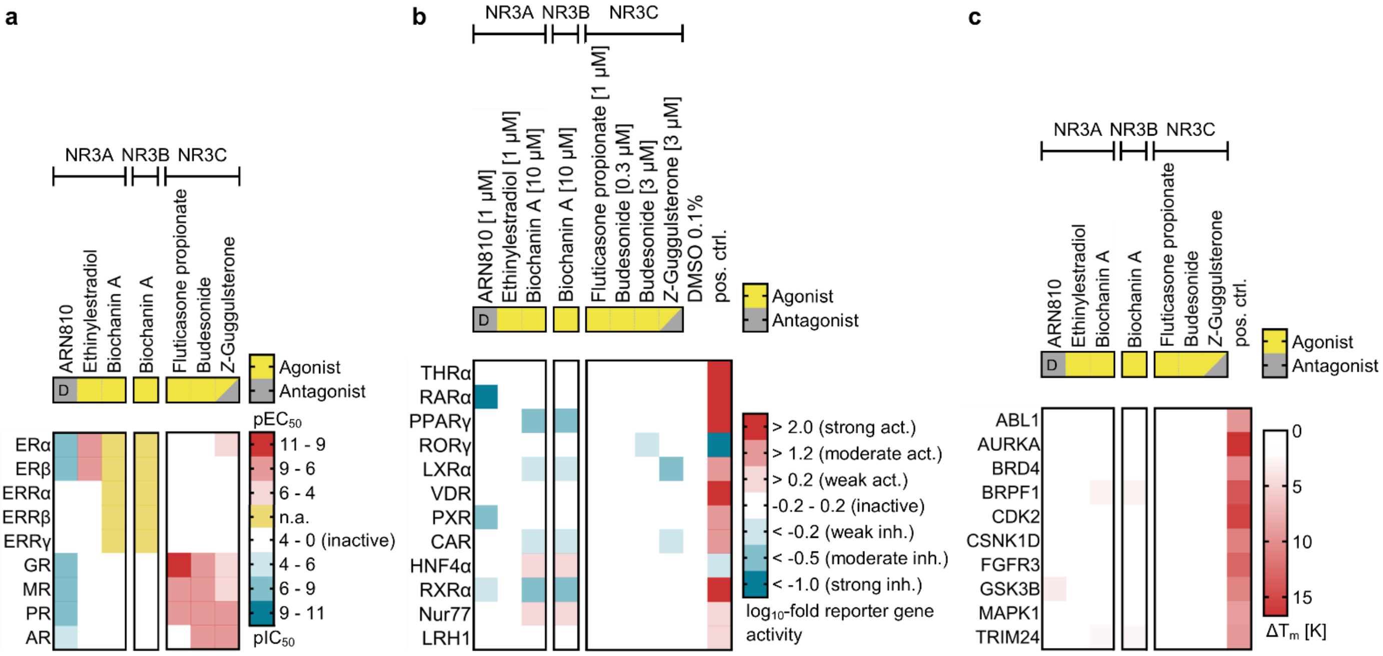

Supplementary Figure 2. Activity and selectivity profiling of the excluded NR3 CG compound candidates. Compounds are labeled according to their mode of action (yellow - agonist, grey - antagonist, grey with label D - degrader) and grouped by their NR3 subfamily main targets. **a** NR3 modulation profiles of the excluded NR3 CG compound candidates reported in literature. The heatmap shows the potency expressed as pEC_{50} (red; agonists) or pIC_{50} (blue; antagonists and inverse agonists) reported in literature. For Biochanin A, only single-point activity data but no EC_{50}/IC_{50} has been reported (yellow; n.a.). **b** Selectivity profiling of the excluded NR3 CG compound candidates at the indicated concentrations in uniform Gal4-hybrid reporter gene assays for selected NRs representing the NR1, NR2, NR4 and NR5 families. As reference ligands T3 (1 µM; NR1A1 (THRα)), retinoic acid (1 µM; NR1B1 (RARα)), rosiglitazone (1 µM; NR1C3 (PPARγ)), SR1001 (1 µM; NR1F3 (RORγ)), T0901317 (1 µM; NR1H3 (LXRα)), calcitriol (1 µM; NR1I1 (VDR)), SR12813 (1 µM; NR1I2 (PXR)), CITCO (1 µM; NRI3 (CAR)), compound 9 from [1] (30 µM; NR2A1 (HNF4α)), bexarotene (1 µM; NR2B1 (RXRα)), amodiaquine (100 µM; NR4A1 (Nur77)) and an in-house compound (50 µM; NR5A2 (LRH1)) were used. **c** Profiling of the excluded NR3 CG compound candidates for off-target binding in a liability target screening by differential scanning fluorimetry (DSF). Proteins were used at 2 µM; staurosporine (ABL1, AURKA, CDK2, FGFR3 and GSK3B), (+)-JQ1 (BRD4), GSK6853 (BRPF1), PK016714a (CSNK1D), GDC-0994 (MAPK1) and IACS-9571 (TRIM24) served as positive controls (pos. ctrl) at a concentration of 20 µM. The heatmap shows the mean ΔT_M calculated by the Boltzmann fit (n=2).

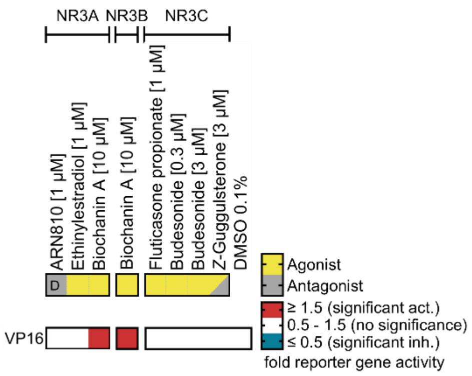

Supplementary Figure 3. Non-specific transcriptional effects of the excluded NR3 CG compound candidates. Compounds are labeled according to their mode of action (yellow - agonist, grey - antagonist, grey with label D - degrader) and grouped by their NR3 subfamily main targets. The heatmap shows compound mediated induction (red) and reduction (blue) of Gal4-VP16 dependent reporter activity at the indicated concentrations expressed as mean fold reporter gene activity (n=3).

Supplementary Table 1. Biochemical properties and recommended concentrations for phenotypic screening of the primary NR3 CG set, containing 40 chemically diverse and selective compounds.

Compound	Reference	Main NR target	Potency [nM] [rel. activity from literature]	Type	NR off-target at recom. conc.	Other off-target at recom. conc.	Recom. conc. or reason f. exclusion	NR3 CG set
**Propylpyrazoletriol (CAS# 263717-53-9)** 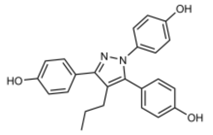	2,3	NR3A1 (ERα)	EC_{50} = 0.1[z] (lit.) [120% of estradiol]	Agonist	-	-	1 µM	Yes
**AZD9496 (CAS# 1639042-08-2)** 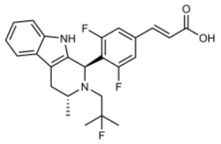	4	NR3A1 (ERα)	IC_{50} = 0.28 ± 0.04[z] (lit.)	Antagonist (Degrader)	NR3C3[4]	-	1 µM	Yes
**Ormeloxifene (CAS# 31477-60-8)** 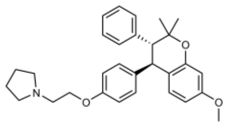	5	NR3A1 (ERα)	EC_{50} = 13.0 ± 0.4[z] (lit.) [25 ± 2% of moxestrol]	Agonist	-	-	1 µM	Yes
**Fulvestrant (CAS# 129453-61-8)** 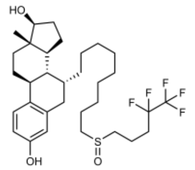	6–8	NR3A1 (ERα) NR3A2 (ERβ)	IC_{50} = 3[z] (lit.) [18% ERα remaining] IC_{50} = 4[z] (lit.) [9% rem. act. of estradiol]	Antagonist (Degrader)	NR1H4[9], NR1I2[9]	sEH[10]	1 µM	Yes
**(*R*,*R*)-THC (CAS# 138090-06-9)** 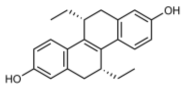	11, 12	NR3A1 (ERα) NR3A2 (ERβ)	EC_{50} = ~4[z] (lit.) [4 ± 1 RBA to estradiol] IC_{50} = ~4[z] (lit.) [25 ± 6 RBA to estradiol]	Agonist, Antagonist	NR1H4[9]	-	1 µM	Yes
**ARN810 (CAS# 1365888-06-7)** 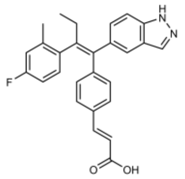	13	NR3A1 (ERα) NR3A2 (ERβ)	IC_{50} = 6[z] (lit.) [9% ERα remaining] IC_{50} = 9[z] (lit.)	Antagonist (Degrader)	NR3C1[13], NR3C2[13], NR3C3[13], NR3C4[13]	$GABA_A$[13], DAT[13]	too many off-targets and better alternatives available	No
**Diarylpropionitrile (CAS# 1428-67-7)** 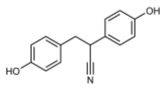	14, 15	NR3A1 (ERα) NR3A2 (ERβ)	EC50 = 66[z] (lit.) EC50 = 0.9[z] (lit.)	Agonist	-	-	1 µM	Yes
**Ethinylestradiol (CAS# 57-63-6)** 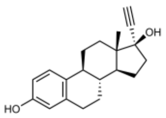	16	NR3A1 (ERα) NR3A2 (ERβ)	EC_{50} = 8 ± 1[x] (lit.) EC50 = 18 ± 2[x] (lit.)	Agonist	NR1I3[17]	AOX1[18], CYP2B6[19], SULT1A1[20], OCT2[21]	too many off-targets and better alternatives available	No

Compound	Reference	Main NR target	Potency [nM] [rel. activity from literature]	Type	NR off-target	Other off-target at recom. conc.	Recom. conc. or reason f. exclusion	NR3 CG set
**Bazedoxifene (CAS# 198481-33-3)** 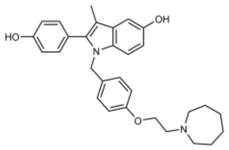	22	NR3A1 (ERα) NR3A2 (ERβ)	EC_{50} = 26 ± 14^{y} (lit.) EC_{50} = 99 ± 53^{y} (lit.)	Antagonist	-	-	1 µM	Yes
**WAY200070 (CAS# 440122-66-7)** 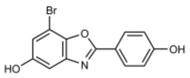	23, 24	NR3A1 (ERα) NR3A2 (ERβ)	EC_{50} = 187 ± 63^{y} (lit.) EC_{50} = 2.0 ± 0.4^{y} (lit.)	Agonist	-	-	1 µM	Yes
**PHTPP (CAS# 805239-56-9)** 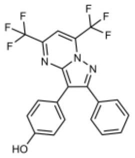	25	NR3A2 (ERβ)	EC_{50} = ~200^{z} (lit.)	Antagonist	NR3A1[25]	-	1 µM	Yes
**Isoliquiritigenin (CAS# 961-29-5)** 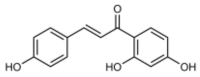	26	NR3A2 (ERβ)	EC_{50} = 269^{z} (lit.)	Agonist	NR3A1[26]	17β-HSD1[27], 17β-HSD2[27], NLRP3[28], MCP[29]	1 µM	Yes
**Biochanin A (CAS# 491-80-5)** 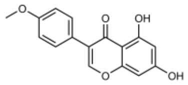	30	NR3A1 (ERα) NR3A2 (ERβ) NR3B1 (ERRα) NR3B2 (ERRβ) NR3B3 (ERRγ)	EC_{50} = n.d. (lit.) [320-fold activation] EC_{50} = n.d. (lit.) [250-fold activation] EC_{50} = n.d. (lit.) [3-fold activation] EC_{50} = n.d. (lit.) [2-fold activation] EC_{50} = n.d. (lit.) [2.3-fold activation]	Agonist	-	CA1[31], CA2[31], CA4[31], CA7[31], CA12[31], CBR1[32], CYP1B1[33], 17β-HSD2[34], 17β-HSD3[35]	too many off-targets	No
**(*Z*)-4-Hydroxytamoxifen (CAS# 68047-06-3)** 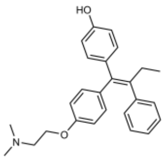	36, 37	NR3A1 (ERα) NR3A2 (ERβ) NR3B2 (ERRβ) NR3B3 (ERRγ)	IC_{50} = 2 ± 1^{y} (lit.) IC_{50} = 1 ± 1^{y} (lit.) IC_{50} = 650^{z} (lit.) IC_{50} = 600^{z} (lit.)	Antagonist, inverse Agonist	-	PLD1[38], PLD2[38]	0.3 µM (NR3A) 3 µM (NR3B)	Yes
**Diethylstilbestrol (CAS# 56-53-1)** 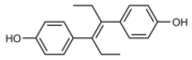	39, 40	NR3A1 (ERα) NR3A2 (ERβ) NR3B3 (ERRγ)	EC_{50} = 0.06^{z} (lit.) EC_{50} = 0.02^{z} (lit.) IC_{50} = 630^{z} (lit.)	Agonist, inverse Agonist	-	AOX1[18]	0.3 µM (NR3A) 3 µM (NR3B)	Yes
**XCT790 (CAS# 725247-18-7)** 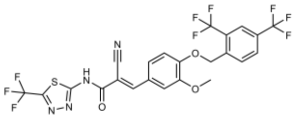	41–44	NR3B1 (ERRα)	IC_{50} = 370^{z} (lit.) [60 – 50% rem. act. at 1 µM]	inverse Agonist	-	-	1 µM	Yes
**GSK4716 (CAS# 101574-65-6)** 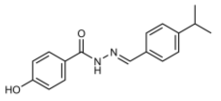	45, 46	NR3B2 (ERRβ) NR3B3 (ERRγ)	EC_{50} = n.d. (lit.) [10-fold activation] EC_{50} = 1899^{z} (lit.) [6-fold activation]	Agonist	-	-	3 µM	Yes

Compound	Reference	Main NR target	Potency [nM] [rel. activity from literature]	Type	NR off-target	Other off-target at recom. conc.	Recom. conc. or reason f. exclusion	NR3 CG set
**DY131 (CAS# 95167-41-2)** 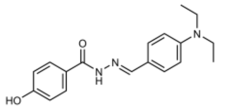	46, 47	NR3B2 (ERRβ) NR3B3 (ERRγ)	EC_{50} = n.d. (lit.) [2-fold activation] EC_{50} = 130[z] (lit.)	Agonist	-	-	3 µM	Yes
**GSK5182 (CAS# 877387-37-6)** 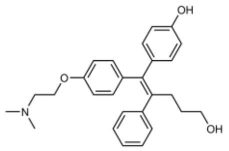	36, 48	NR3B2 (ERRβ) NR3B3 (ERRγ)	EC_{50} = 3500[z] (lit.) EC_{50} = 2700[z] (lit.) [31% rem. act. at 1 µM]	inverse Agonist	NR3A1[48]	-	10 µM	Yes
**Bisphenol A (CAS# 80-05-7)** 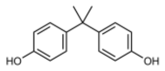	49	NR3B3 (ERRγ)	IC_{50} = 13 ± 2[z] (lit.)	inverse Antagonist	NR3A1[50], NR3A2[50], NR3C4[51]	-	0.3 µM	Yes
**GSK9027 (CAS# 1229096-88-1)** 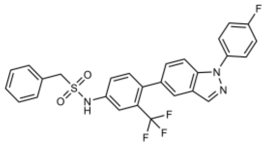	52	NR3C1 (GR)	EC_{50} = 10 ± 5[y] (lit.) [102 ± 7% of dexamet.]	Agonist	-	-	1 µM	Yes
**AZD5423 (CAS# 1034148-04-3)** 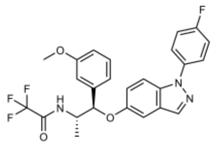	53, 54	NR3C1 (GR)	EC_{50} = 0.9 ± 0.2[y] (lit.)	Agonist	NR3C2[53], NR3C3[53]	-	1 µM	Yes
**Beclomethasone (CAS# 4419-39-0)** 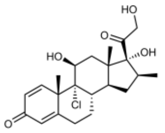	55	NR3C1 (GR)	EC_{50} = 8[z] (lit.)	Agonist	-	-	1 µM	Yes
**Mapracorat (CAS# 887375-26-0)** 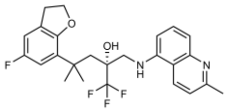	56	NR3C1 (GR)	EC_{50} = 1.6 ± 0.3[y] (lit.) [89 ± 4% transrepression]	Agonist	-	-	1 µM	Yes
**Fludrocortisone acetate (CAS# 514-36-3)** 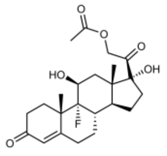	57	NR3C1 (GR) NR3C2 (MR)	EC_{50} = 4[z] (lit.) [~1000-fold activation] EC_{50} = 0.2[z] (lit.) [~41-fold activation]	Agonist	-	-	1 µM	Yes
**Fluticasone propionate (CAS# 80474-14-2)** 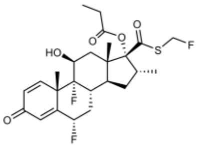	58, 59	NR3C1 (GR) NR3C2 (MR) NR3C3 (PR)	EC_{50} = 1[z] (lit.) EC_{50} = 149 ± 166[y] (lit.) EC_{50} = 21 ± 14[y] (lit.)	Agonist	-	-	More potent and selective alternatives	No

Compound	Reference	Main NR target	Potency [nM] [rel. activity from literature]	Type	NR off-target	Other off-target at recom. conc.	Recom. conc. or reason f. exclusion	NR3 CG set
**Mifepristone (CAS# 84371-65-3)**<br>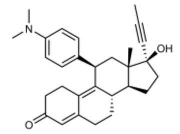	60–65	NR3C1 (GR)<br>NR3C2 (MR)<br>NR3C3 (PR)<br>NR3C4 (AR)	$IC_{50} = 0.8 \pm 0.1^{x}$ (lit.)<br>[5 ± 1% rem. act. of DXM)<br>$IC_{50} = 590^{z}$ (lit.)<br>[4% rem. act.]<br>$IC_{50} = 0.18 \pm 0.02^{x}$ (lit.)<br>[4 ± 1% rem. act. of PGN)<br>$IC_{50} = 5 \pm 2^{x}$ (lit.)<br>[25 ± 2% rem. act. of DHT)	Antagonist	NR3A2[60–65]	CYP2C8[66,67],<br>CYP2C9[66],<br>CYP3A4[66,67],<br>BSEP[68]	3 µM	Yes
**Methyltrienolone (CAS# 965-93-5)**<br>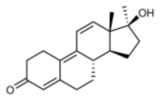	69	NR3C1 (GR)<br>NR3C2 (MR)<br>NR3C3 (PR)<br>NR3C4 (AR)	$EC_{50} = 10 \pm 4^{y}$ (lit.)<br>$EC_{50} = 0.5 \pm 0.1^{y}$ (lit.)<br>$EC_{50} = 0.5 \pm 0.3^{y}$ (lit.)<br>$EC_{50} = 0.01 \pm 0.01^{y}$ (lit.)	Agonist	-	-	1 µM	Yes
**Medroxyprogesterone (CAS# 520-85-4)**<br>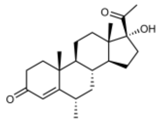	70–76	NR3C1 (GR)<br>NR3C3 (PR)<br>NR3C4 (AR)	$EC_{50} = 10 \pm 1^{x}$ (lit.)<br>[157 ± 22% of DXM]<br>$EC_{50} = 0.15 \pm 0.05^{x}$ (lit.)<br>[80 ± 7% of PGN]<br>$IC_{50} = 6 \pm 1^{x}$ (lit.)<br>[159 ± 10% of DHT]	Agonist	NR3A1[74–76],<br>NR3C2[74–76]	17β-HSD1[77],<br>17β-HSD2[77],<br>17β-HSD3[77]	0.3 µM	Yes
**Budesonide (CAS# 51333-22-3)**<br>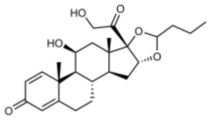	58, 59	NR3C1 (GR)<br>NR3C2 (MR)<br>NR3C3 (PR)<br>NR3C4 (AR)	$EC_{50} = 12^{z}$ (lit.)<br>$EC_{50} = 14 \pm 10^{y}$ (lit.)<br>$EC_{50} = 28 \pm 7^{y}$ (lit.)<br>$EC_{50} = 710 \pm 170^{y}$ (lit.)	Agonist	-	-	More potent alternative	No
**Aldosterone (CAS# 52-39-1)**<br>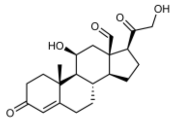	78	NR3C2 (MR)	$EC_{50} = 0.34 \pm 0.04^{x}$ (lit.)	Agonist	-	-	1 µM	Yes
**Eplerenone (CAS# 107724-20-9)**<br>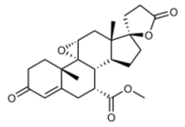	79–81	NR3C2 (MR)	$IC_{50} = 122^{z}$ (lit.)	Antagonist	-	-	1 µM	Yes
**PF-03882845 (CAS# 1023650-66-9)**<br>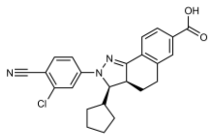	81	NR3C2 (MR)<br>NR3C3 (PR)	$EC_{50} = 9^{z}$ (lit.)<br>$EC_{50} = 416^{z}$ (lit.)	Antagonist	-	-	1 µM	Yes
***Z*-Guggulsterone (CAS# 39025-23-5)**<br>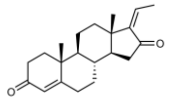	82	NR3C3 (PR)<br>NR3C4 (AR)	$IC_{50} = 740 \pm 220^{x}$ (lit.)<br>$EC_{50} = 660 \pm 240^{x}$ (lit.)	Antagonist, Agonist	NR1H4[82],<br>NR1I2[82],<br>NR3A1[82],<br>NR3C1[82],<br>NR3C2[82]	-	Too many off-targets	No
**Ulipristal acetate (CAS# 126784-99-4)**<br>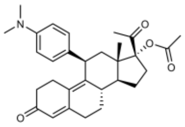	83	NR3C3 (PR)	$EC_{50} = >100^{z}$ (lit.)	Agonist (Modulator)	-	-	1 µM	Yes

Compound	Reference	Main NR target	Potency [nM] [rel. activity from literature]	Type	NR off-target	Other off-target at recom. conc.	Recom. conc. or reason f. exclusion	NR3 CG set
**Andarine (CAS# 401900-40-1)** 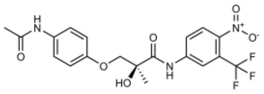	84–86	NR3C4 (AR)	EC_{50} = 4.0 ± 0.7^{x} (lit.) [93 ± 7% of DHT]	Agonist	-	-	1 µM	Yes
**PF-998425 (CAS# 1076225-27-8)** 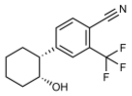	87	NR3C4 (AR)	IC_{50} = 43 ± 12^{x} (lit.)	Antagonist	-	-	1 µM	Yes
**Cyproterone acetate (CAS# 427-51-0)** 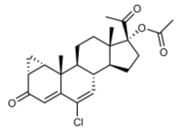	88	NR3C4 (AR)	IC_{50} = 26 ± 23^{x} [52 ± 6% rem. act. of DHT]	Antagonist	-	-	1 µM	Yes
**Enzalutamide (CAS# 915087-33-1)** 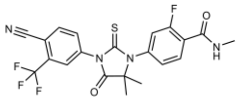	89	NR3C4 (AR)	IC_{50} = 361^{z} (lit.) [5% rem. act. of R1881]	Antagonist	-	-	1 µM	Yes
**BMS564929 (CAS# 627530-84-1)** 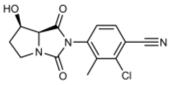	90, 91	NR3C4 (AR)	EC_{50} = 0.7^{z} (lit.)	Agonist	-	-	1 µM	Yes

^{x} mean ± S.E.M.
^{y} mean ± SD
^{z} information on error or error type not available
RBA relative binding affinity
DXM dexamethasone
PGN progesterone
DHT dihydrotestosterone
sEH soluble epoxid hydrolase
DAT dopamine transporter
AOX1 aldehyde oxidase 1
CYP cytochrome P450
SULT1A1 sulfonyltransferase 1A1
OCT2 organic cation transporter 2
17β-HSD 17β-hydroxysteroid dehydrogenase
NLRP3 nod-like receptor 3
MCP multi-catalytic protease
CA carbonic anhydrase
CBRP1 carbonyl reductase 1
PLD phospholipase D
BSEP bile salt export pump

Supplementary Table 2. Off-targets for the excluded NR3 CG compound candidates ARN810, Ethinylestradiol, Biochanin A and *Z*-Guggulsterone with annotated bioactivity values ≤ 10 µM from previously published dataset[92].

Compound	Off-targets ≤ 10 µM	p(Potency)	Type	Compound	Off-targets ≤ 10 µM	p(Potency)	Type
**ARN810**	NR3C1	6.00	Antagonist	**Ethinylestradiol**	NR1I3	5.52	inv. Agonist
	NR3C2	6.00	Antagonist		AOX1	6.24	Inhibitor
	NR3C3	6.00	Antagonist		CYP2B6	6.05	Inhibitor
	NR3C4	5.40	Antagonist		SULT1A1	7.70	Inhibitor
	$GABA_A$	5.96	Inhibitor		OCT2	5.66	Inhibitor
	DAT	5.47	Inhibitor				
**Biochanin A**	CA1	5.00	Antagonist	***Z*-Guggulsterone**	NR1H4	4.83	Antagonist
	CA2	5.00	Antagonist		NR1I2	8.62	Agonist
	CA4	5.15	Antagonist		NR3A1	5.30	Agonist
	CA7	6.43	Antagonist		NR3C1	5.22	Antagonist
	CA12	7.28	Antagonist		NR3C2	5.73	Antagonist
	CBR1	4.90	Antagonist				
	CYP1B1	5.72	Inhibitor				
	17β-HSD2	5.00	Inhibitor				
	17β-HSD3	4.97	Inhibitor				

DAT dopamine transporter
CA carbonic anhydrase
CBRP1 carbonyl reductase 1
17β-HSD 17β-hydroxysteroid dehydrogenase
AOX1 aldehyde oxidase 1
SULT1A1 sulfonyltransferase 1A1
OCT2 organic cation transporter 2

Supplementary Table 3. NR3 CG compound candidates with annotated bioactivity data ≤ 10 µM and commercial availability from previously published dataset[92]. Stepwise compound selection as described in Fig. 2.

No.	CG compound candidate	CAS-No.	Smiles	Reason for exclusion
1	G15	1161002-05-6	BrC1=CC(OCO2)=C2C=C1[C@H](NC3=CC=CC=C43)[C@@H]5[C@H]4C=CC5	> 1 µM
2	Danazol	17230-88-5	C#C[C@@]1(CC[C@H]2[C@@H]3CCC4=Cc5c(C[C@@]4([C@H]3CC[C@@]21C)C)cno5)O	> 1 µM
3	Kurarinone	34981-26-5	C/C(C)=C\C[C@@H](C(C)=C)CC1=C2C(C(C[C@@H](C3=C(O)C=C(O)C=C3)O2)=O)=C(OC)C=C1O	> 1 µM
4	Calcitriol	32222-06-3	C[C@@H]([C@H]1CC[C@@H]2[C@]1(C)CCC/C2=C\C=C3C[C@@H](O)C[C@H](O)C\3=C)CCCC(C)(O)C	> 1 µM
5	AZD3514	1240299-33-5	CC(N1CCN(CCOC2=CC=C(C3CCN(C4=NN5C(CC4)=NN=C5C(F)(F)F)CC3)C=C2)CC1)=O	> 1 µM
6	Cortisone acetate	50-04-4	CC(OCC([C@]1(CC[C@H]2[C@@H]3CCC4=CC(CC[C@@]4([C@H]3C(C[C@@]21C)=O)C)=O)O)=O)=O	> 1 µM
7	Pregnenolone carbonitrile	1434-54-4	CC([C@H]1[C@@H](C[C@H]2[C@@H]3CC=C4C[C@H](CC[C@@]4([C@H]3CC[C@@]21C)C)O)C#N)=O	> 1 µM
8	G-1	881639-98-1	CC(C1=CC2=C(N[C@@H](C3=CC4=C(OCO4)C=C3Br)[C@@H]5[C@H]2C=CC5)C=C1)=O	> 1 µM
9	XL335	629664-81-9	CC(OC(C1=CN(C(C2=CC(F)=C(F)C=C2)=O)CC(C)(C)C3=C1NC4=CC=CC=C43)=O)C	> 1 µM
10	Octyl gallate	1034-01-1	CCCCCCCCOC(C1=CC(O)=C(O)C(O)=C1)=O	> 1 µM
11	Butylparaben	94-26-8	CCCCOC(C1=CC=C(O)C=C1)=O	> 1 µM
12	BRD4354	315698-07-8	CCN1CCN(C(C2=CC(Cl)=C3C=CC=NC3=C2O)C4=CN=CC=C4)CC1	> 1 µM
13	Ethylparaben	120-47-8	CCOC(C1=CC=C(O)C=C1)=O	> 1 µM
14	Isoxanthohumol	521-48-2	C/C(C)=C\CC1=C2C(C(CC(C3=CC=C(O)C=C3)O2)=O)=C(OC)C=C1O	> 1 µM
15	5-Methoxyflavone	42079-78-7	COC1=CC=CC2=C1C(C=C(C3=CC=CC=C3)O2)=O	> 1 µM
16	2'-Hydroxy-2-methoxychalcone	42220-77-9	COC1=CC=CC=C1/C=C/C(C2=CC=CC=C2O)=O	> 1 µM
17	WAY-639019	793728-59-3	COC1=CC=CC=C1N2CCN(C(C3=CC4=C(CCC4)S3)=O)CC2	> 1 µM
18	*β*-Zearalenol	71030-11-0	C[C@H]1CCC[C@@H](O)CCC/C=C/C2=C(C(O1)=O)C(O)=CC(O)=C2	> 1 µM
19	*17α*-Methyltestosterone	58-18-4	C[C@@]1(CC[C@H]2[C@@H]3CCC4=CC(CC[C@@]4([C@H]3CC[C@@]21C)C)=O)O	> 1 µM
20	Emodin	518-82-1	CC1=CC2=C(C(C3=C(C=C(O)C=C3O)C2=O)=O)C(O)=C1	> 1 µM
21	Gossypol	303-45-7	CC1=CC2=C(C(O)=C1C3=C(O)C4=C(C(C(C)C)=C(O)C(O)=C4C=O)C=C3C)C(C=O)=C(O)C(O)=C2C(C)C	> 1 µM
22	Clotrimazole	23593-75-1	ClC1=CC=CC=C1C(C2=CC=CC=C2)(N3C=CN=C3)C4=CC=CC=C4	> 1 µM
23	LY2562175	1103500-20-4	CN1C=C(C(O)=O)C2=C1C=C(N3CCC(OCC4=C(C5CC5)ON=C4C6=C(Cl)C=CC=C6Cl)CC3)C=C2	> 1 µM
24	LXR-623	875787-07-8	FC(F)(F)C1=CC=CC2=C(C3=CC=C(F)C=C3)N(CC4=C(Cl)C=C(F)C=C4)N=C21	> 1 µM
25	Caffeic acid phenylethylester	104594-70-9	O=C(/C=C/C1=CC(O)=C(O)C=C1)OCCC2=CC=CC=C2	> 1 µM
26	WAY-273170	478247-52-8	O=C(NC(SCC(C1=CC=C(Cl)C=C1)=O)=N2)C(C=N3)=C2N3C4=CC=CC=C4	> 1 µM
27	Coumaric acid	91-64-5	O=C1C=CC2=CC=CC=C2O1	> 1 µM

No.	CG compound candidate	CAS-No.	Smiles	Reason for exclusion
28	WAY-605337	485362-61-6	O=C(COC(C1=CC(C2=CC=CO2)=NC3=CC=CC=C31)=O)N4CCN(C(C5=CC=CO5)=O)CC4	> 1 μM
29	5-hydroxy-1-naphthyl benzoate	2444-20-4	O=C(OC1=CC=CC2=C1C=CC=C2O)C3=CC=CC=C3	> 1 μM
30	Kaempferol	520-18-3	O=C(C1=C(O)C=C(O)C=C1O2)C(O)=C2C3=CC=C(O)C=C3	> 1 μM
31	Morin	480-16-0	OC1=CC(O)=C(C2=C(O)C(C3=C(O)C=C(O)C=C3O2)=O)C=C1	> 1 μM
32	Apigenin	520-36-5	O=C(C1=C(O)C=C(O)C=C1O2)C=C2C3=CC=C(O)C=C3	> 1 μM
33	Flavone	525-82-6	O=C(C1=CC=CC=C1O2)C=C2C3=CC=CC=C3	> 1 μM
34	Bithionol	97-18-7	ClC1=CC(SC2=C(O)C(Cl)=CC(Cl)=C2)=C(O)C(Cl)=C1	> 1 μM
35	WAY-323877	167483-31-0	C1(CCSC2=NC3=CC=CC=C3N2)=CC=CC=C1	> 1 μM
36	Ethinylestradiol	57-63-6	C[C@]12CC[C@H]3[C@@H](CCC4=C3C=CC(O)=C4)[C@@H]1CC[C@@]2(O)C#C	> 5 off-targets
37	*E*-Guggulsterone	39025-24-6	C/C=C1C(C[C@H]2[C@@H]3CCC4=CC(CC[C@@]4([C@H]3CC[C@]/12C)C)=O)=O	> 5 off-targets
38	*Z*-Guggulsterone	39025-23-5	C/C=C1C(C[C@@H]2[C@]\1(C)CC[C@H]3[C@H]2CCC4=CC(CC[C@]34C)=O)=O	> 5 off-targets
39	Biochanin A	491-80-5	COC1=CC=C(C2=COC3=CC(O)=CC(O)=C3C2=O)C=C1	> 5 off-targets
40	Progesterone	57-83-0	CC(C1CCC2C3CCC4=CC(CCC4(C3CCC12C)C)=O)=O	> 5 off-targets
41	Medroxyprogesterone acetate	71-58-9	CC(O[C@@]1(CC[C@H]2[C@@H]3C[C@@H](C4=CC(CC[C@@]4([C@H]3CC[C@@]21C)C)=O)C)C(C)=O)=O	> 5 off-targets
42	Spironolactone	52-01-7	CC(S[C@@H]1CC2=CC(CC[C@@]2([C@H]3CC[C@]4([C@H]([C@@H]31)CC[C@@]45CCC(O5)=O)C)C)=O)=O	> 5 off-targets
43	Hydroxyprogesterone	68-96-2	CC([C@]1(CC[C@H]2[C@@H]3CCC4=CC(CC[C@@]4([C@H]3CC[C@@]21C)C)=O)O)=O	> 5 off-targets
44	Flutamide	13311-84-7	CC(C(NC1=CC(C(F)(F)F)=C([N+]([O-])=O)C=C1)=O)C	> 5 off-targets
45	Bicalutamide	90357-06-5	CC(C(NC1=CC(C(F)(F)F)=C(C#N)C=C1)=O)(O)CS(=O)(C2=CC=C(F)C=C2)=O	> 5 off-targets
46	ARN810	1365888-06-7	CC/C(C1=C(Cl)C=C(F)C=C1)=C(C2=CC3=C(NN=C3)C=C2)/C4=CC=C(/C=C/C(O)=O)C=C4	> 5 off-targets
47	(*E,Z*)-Hydroxytamoxifen	68392-35-8	CC/C(C1=CC=CC=C1)=C(C2=CC=C(OCCN(C)C)C=C2)\C3=CC=C(O)C=C3	> 5 off-targets
48	Tamoxifen	10540-29-1	CC/C(C1=CC=CC=C1)=C(C2=CC=C(OCCN(C)C)C=C2)\C3=CC=CC=C3	> 5 off-targets
49	Prednisolone	50-24-8	C[C@]12C[C@@H]([C@H]3[C@H]([C@@H]1CC[C@@]2(C(CO)=O)O)CCC4=CC(C=C[C@@]43C)=O)O	> 5 off-targets
50	Dihydrotestosterone	521-18-6	C[C@]12CC[C@H]3[C@H]([C@@H]1CC[C@@H]2O)CC[C@H]4CC(CC[C@@]43C)=O	> 5 off-targets
51	Estrone	53-16-7	C[C@]12CC[C@H]3[C@@H](CCC4=C3C=CC(O)=C4)[C@@H]1CCC2=O	> 5 off-targets
52	*17β*-Estradiol	50-28-2	C[C@]12CC[C@H]3[C@@H](CCC4=C3C=CC(O)=C4)[C@@H]1CC[C@@H]2O	> 5 off-targets
53	Fluticasone propionate	80474-14-2	CCC(O[C@@]1([C@@H](C[C@H]2[C@@H]3C[C@@H](C4=CC(C=C[C@@]4([C@]3([C@H](C[C@@]21C)O)F)C)=O)F)C)C(SCF)=O)=O	> 5 off-targets
54	Budesonide	51333-22-3	CCCC1O[C@@H]2C[C@H]3[C@@H]4CCC5=CC(C=C[C@@]5([C@H]4[C@H](C[C@@]3([C@@]2(O1)C(CO)=O)C)O)C)=O	> 5 off-targets

No.	CG compound candidate	CAS-No.	Smiles	Reason for exclusion
55	Enclomiphene hydrochloride	14158-65-7	CCN(CCOC1=CC=C(/C(C2=CC=CC=C2)=C(Cl)\C3=CC=CC=C3)C=C1)CC.C	> 5 off-targets
56	Podophyllotoxin	518-28-5	COC1=CC([C@H]2[C@@H]3[C@@H]([C@@H](O)C4=CC5=C(OCO5)C=C24)COC3=O)=CC(OC)=C1OC	> 5 off-targets
57	(*E*)-Resveratrol	501-36-0	OC1=CC(/C=C/C2=CC=C(O)C=C2)=CC(O)=C1	> 5 off-targets
58	(*Z*)-3,4',5-Trimethoxystilbene	94608-23-8	COC1=CC=C(/C=C\C2=CC(OC)=CC(OC)=C2)C=C1	> 5 off-targets
59	(*E*)-3,4',5-Trimethoxystilbene	22255-22-7	COC1=CC=C(/C=C/C2=CC(OC)=CC(OC)=C2)C=C1	> 5 off-targets
60	Indomethacin	53-86-1	CC1=C(CC(O)=O)C2=C(C=CC(OC)=C2)N1C(C3=CC=C(Cl)C=C3)=O	> 5 off-targets
61	Fluticasone furoate	397864-44-7	C[C@@H]1C[C@H]2[C@@H]3C[C@H](F)C4=CC(C=C[C@]4(C)[C@@]3(F)[C@@H](O)C[C@]2(C)[C@@]1(OC(C5=CC=CO5)=O)C(SCF)=O)=O	> 5 off-targets
62	Estriol	50-27-1	C[C@]12CC[C@H]3[C@@H](CCC4=C3C=CC(O)=C4)[C@@H]1C[C@@H](O)[C@@H]2O	> 5 off-targets
63	Estradiol benzoate	50-50-0	C[C@]12CC[C@H]3[C@@H](CCC4=C3C=CC(OC(C5=CC=CC=C5)=O)=C4)[C@@H]1CC[C@@H]2O	> 5 off-targets
64	Galeterone	851983-85-2	C[C@]12CC[C@H](O)CC1=CC[C@@H]3[C@@H]2CC[C@@]4(C)[C@H]3CC=C4N5C=NC6=CC=CC=C65	> 5 off-targets
65	Testosterone	58-22-0	C[C@]12CC[C@H]3[C@H]([C@@H]1CC[C@@H]2O)CCC4=CC(CC[C@@]43C)=O	> 5 off-targets
66	Triamcinolone	124-94-7	C[C@]12C[C@@H]([C@]3([C@H]([C@@H]1C[C@H]([C@@]2(C(CO)=O)O)O)CCC4=CC(C=C[C@@]43C)=O)F)O	> 5 off-targets
67	Raloxifene	84449-90-1	O=C(C1=C(C2=CC=C(O)C=C2)SC3=C1C=CC(O)=C3)C(C=C4)=CC=C4OCCN5CCCCC5	> 5 off-targets
68	Genistein	446-72-0	O=C1C2=C(O)C=C(O)C=C2OC=C1C3=CC=C(O)C=C3	> 5 off-targets
69	Daidzein	486-66-8	O=C1C(C=CC(O)=C2)=C2OC=C1C3=CC=C(O)C=C3	> 5 off-targets
70	Coumestrol	479-13-0	OC1=CC(OC2=C3C(OC4=C2C=CC(O)=C4)=O)=C3C=C1	> 5 off-targets
71	Hexachlorophene	70-30-4	ClC1=C(Cl)C(CC2=C(O)C(Cl)=CC(Cl)=C2Cl)=C(O)C(Cl)=C1	> 5 off-targets
72	GSK4716	101574-65-6	CC(C1=CC=C(/C=N/NC(C2=CC=C(O)C=C2)=O)C=C1)C	
73	Levonorgestrel	6533-00-2	C#CC1(CC[C@H]2[C@@H]3CCC4=CC(CC[C@@H]4[C@H]3CC[C@@]21CC)=O)O	
74	Ethisterone	434-03-7	C#C[C@@]1(CC[C@H]2[C@@H]3CCC4=CC(CC[C@@]4([C@H]3CC[C@@]21C)C)=O)O	
75	GSK5182	877387-37-6	CN(CCOC1=CC=C(/C(C2=CC=C(O)C=C2)=C(C3=CC=CC=C3)/CCCO)C=C1)C	
76	Norethisterone	68-22-4	C#C[C@@]1(CC[C@H]2[C@@H]3CCC4=CC(CC[C@@H]4[C@H]3CC[C@@]21C)=O)O	
77	Norethisterone acetate	51-98-9	C#C[C@@]1(CC[C@H]2[C@@H]3CCC4=CC(CC[C@@H]4[C@H]3CC[C@@]21C)=O)OC(C)=O	
78	Mestranol	72-33-3	C[C@]12CC[C@H]3[C@@H](CCC4=C3C=CC(OC)=C4)[C@@H]1CC[C@@]2(O)C#C	
79	Tibolone	5630-53-5	C#C[C@]1(O)CC[C@H]2[C@H]3[C@H](CC[C@@]21C)C4=C(CC(CC4)=O)C[C@H]3C	
80	ERB 041	524684-52-4	C=CC1=C2C(N=C(C3=CC(F)=C(O)C=C3)O2)=CC(O)=C1	
81	Mifepristone	84371-65-3	CC#C[C@]1(O)CC[C@@H]2[C@]1(C)C[C@H](C3=CC=C(N(C)C)C=C3)C4=C5CCC(C=C5CC[C@@H]24)=O	

No.	CG compound candidate	CAS-No.	Smiles	Reason for exclusion
82	Andarine	401900-40-1	CC(NC1=CC=C(OC[C@@](C(NC2=CC(C(F)(F)F)=C([N+]([O-])=O)C=C2)=O)(O)C)C=C1)=O	
83	Ulipristal	159811-51-5	CC(OC1(CCC2C3CCC4=CC(CCC4=C3C(CC21C)c5ccc(N(C)C)cc5)=O)C(C)=O)=O	
84	Fludrocortisone acetate	514-36-3	CC(OCC([C@]1(CC[C@H]2[C@@H]3CCC4=CC(CC[C@@]4([C@]3([C@H](C[C@@]21C)O)F)C)=O)O)=O)=O	
85	Diflorasone diacetate	33564-31-7	CC(OCC([C@]1([C@H](C[C@H]2[C@@H]3C[C@@H](C4=CC(C=C[C@@]4([C@]3([C@H](C[C@@]21C)O)F)C)=O)F)C)OC(C)=O)=O)=O	
86	Fluocinonide	356-12-7	CC(OCC([C@@]12OC(C)(O[C@@H]1C[C@H]3[C@@H]4C[C@@H](C5=CC(C=C[C@@]5([C@]4([C@H](C[C@@]32C)O)F)C)=O)F)C)=O)=O	
87	Megestrol acetate	595-33-5	CC(O[C@@]1(CC[C@H]2[C@@H]3C=C(C4=CC(CC[C@@]4([C@H]3CC[C@@]21C)C)=O)C)C(C)=O)=O	
88	Chlormadinone acetate	302-22-7	CC(O[C@@]1(CC[C@H]2[C@@H]3C=C(C4=CC(CC[C@@]4([C@H]3CC[C@@]21C)C)=O)Cl)C(C)=O)=O	
89	Cyproterone acetate	427-51-0	CC(O[C@@]1(CC[C@H]2[C@@H]3C=C(C4=CC([C@@H]5C[C@@H]5[C@@]4([C@H]3CC[C@@]21C)C)=O)Cl)C(C)=O)=O	
90	AZD5423	1034148-04-3	C[C@H](NC(C(F)(F)F)=O)[C@H](OC1=CC2=C(N(C3=CC=C(F)C=C3)N=C2)C=C1)C4=CC(OC)=CC=C4	
91	Ulipristal acetate	126784-99-4	CC(O[C@@]1(CC[C@H]2[C@@H]3CCC4=CC(CCC4=C3[C@H](C[C@@]21C)c5ccc(N(C)C)cc5)=O)C(C)=O)=O	
92	Cyclofenil	2624-43-3	CC(OC1=CC=C(/C(C2=CC=C(OC(C)=O)C=C2)=C3CCCCC\3)C=C1)=O	
93	Fluorometholone	426-13-1	CC([C@]1(CC[C@H]2[C@@H]3C[C@@H](C4=CC(C=C[C@@]4([C@]3([C@H](C[C@@]21C)O)F)C)=O)C)O)=O	
94	Medroxyprogesterone	520-85-4	CC([C@]1(CC[C@H]2[C@@H]3C[C@@H](C4=CC(CC[C@@]4([C@H]3CC[C@@]21C)C)=O)C)O)=O	
95	Hydroxyflutamide	52806-53-8	CC(C(NC1=CC(C(F)(F)F)=C([N+]([O-])=O)C=C1)=O)(O)C	
96	Bisphenol A	80-05-7	CC(C1=CC=C(O)C=C1)(C2=CC=C(O)C=C2)C	
97	Mapracorat	887375-26-0	CC1=NC2=C(C(NC[C@](C(F)(F)F)(O)CC(C)(C3=CC(F)=CC4=C3OCC4)C)=CC=C2)C=C1	
98	Sophoraflavanone B	53846-50-7	C/C(C)=C\CC1=C2C(C(C[C@@H](C3=CC=C(O)C=C3)O2)=O)=C(O)C=C1O	
99	(*2R/S*)-6-PNG	68682-01-9	C/C(C)=C\CC1=C(O)C2=C(OC(C3=CC=C(O)C=C3)CC2=O)C=C1O	
100	Y134	849662-80-2	CC(N1CCN(C2=CC=C(C(C3=C(C4=CC=C(O)C=C4)SC5=C3C=CC(O)=C5)=O)C=C2)CC1)C	
101	Triptophenolide	74285-86-2	CC(C1=C(O)C2=C([C@@]3(C)CCC4=C(COC4=O)[C@@H]3CC2)C=C1)C	
102	Diethylstilbestrol	56-53-1	CC/C(C1=CC=C(O)C=C1)=C(C2=CC=C(O)C=C2)/CC	
103	(*Z*)-Hydroxytamoxifen	68047-06-3	CC/C(C1=CC=CC=C1)=C(C2=CC=C(OCCN(C)C)C=C2)\C3=CC=C(O)C=C3	
104	4'-Hydroxytamoxifen	82413-23-8	CC/C(C1=CC=C(O)C=C1)=C(C2=CC=C(OCCN(C)C)C=C2)\C3=CC=CC=C3	
105	RU58841	154992-24-2	CC1(C)C(N(C2=CC(C(F)(F)F)=C(C#N)C=C2)C(N1CCCCO)=O)=O	
106	Nilutamide	63612-50-0	CC1(C)C(N(C2=CC(C(F)(F)F)=C([N+]([O-])=O)C=C2)C(N1)=O)=O	
107	Triamcinolone acetonide	76-25-5	CC1(O[C@@H]2C[C@H]3[C@@H]4CCC5=CC(C=C[C@@]5([C@]4([C@H](C[C@@]3([C@@]2(O1)C(CO)=O)C)O)F)C)=O)C	

No.	CG compound candidate	CAS-No.	Smiles	Reason for exclusion
108	Halcinonide	3093-35-4	CC1(O[C@@H]2C[C@H]3[C@@H]4CCC5=CC(CC[C@@]5([C@]4([C@H](C[C@@]3([C@@]2(O1)C(CCl)=O)C)O)F)C)=O)C	
109	Fluocinolone acetonide	67-73-2	CC1(O[C@@H]2C[C@H]3[C@@H]4C[C@@H](C5=CC(C=C[C@@]5([C@]4([C@H](C[C@@]3([C@@]2(O1)C(CO)=O)C)O)F)C)=O)F)C	
110	Flunisolide	3385-03-3	CC1(O[C@@H]2C[C@H]3[C@@H]4C[C@@H](C5=CC(C=C[C@@]5([C@H]4[C@H](C[C@@]3([C@@]2(O1)C(CO)=O)C)O)C)=O)F)C	
111	Flurandrenolide	1524-88-5	CC1(O[C@@H]2C[C@H]3[C@@H]4C[C@@H](C5=CC(CC[C@@]5([C@H]4[C@H](C[C@@]3([C@@]2(O1)C(CO)=O)C)O)C)=O)F)C	
112	Fulvestrant	129453-61-8	C[C@]12CC[C@H]3[C@@H]([C@H](CCCCCCCCCS(CCCC(F)(F)C(F)(F)F)=O)CC4=C3C=CC(O)=C4)[C@@H]1CC[C@@H]2O	
113	Acolbifene	182167-02-8	CC1=C(C2=CC=C(O)C=C2)[C@H](C3=CC=C(OCCN4CCCCC4)C=C3)OC5=C1C=CC(O)=C5	
114	Dexamethasone	50-02-2	C[C@@H]1C[C@H]2[C@@H]3CCC4=CC(C=C[C@@]4([C@]3([C@H](C[C@@]2([C@]1(C(CO)=O)O)C)O)F)C)=O	
115	Hexestrol	84-16-2	CC[C@@H]([C@H](C1=CC=C(O)C=C1)CC)C2=CC=C(O)C=C2	
116	ZK164015	177583-70-9	CCCCCS(=O)(CCCCCCCCCCN1C2=C(C(C)=C1C3=CC=C(O)C=C3)C=C(O)C=C2)=O	
117	Propylpyrazoletriol	263717-53-9	CCCC1=C(C2=CC=C(O)C=C2)N(C3=CC=C(O)C=C3)N=C1C4=CC=C(O)C=C4	
118	DY131	95167-41-2	CCN(C1=CC=C(/C=N/NC(C2=CC=C(O)C=C2)=O)C=C1)CC	
119	(*R,R*)-THC	138090-06-9	CC[C@@H]1CC2=C(C3=C1C4=C(C=C(O)C=C4)C[C@H]3CC)C=CC(O)=C2	
120	Enzalutamide	915087-33-1	CC1(C)C(N(C2=CC(C(F)(F)F)=C(C#N)C=C2)C(N1C3=CC(F)=C(C(NC)=O)C=C3)=S)=O	
121	Apalutamide	956104-40-8	CNC(C1=C(F)C=C(N2C(N(C3=CC(C(F)(F)F)=C(C#N)N=C3)C(C24CCC4)=O)=S)C=C1)=O	
122	Eplerenone	107724-20-9	COC([C@@H]1CC2=CC(CC[C@@]2([C@@]34O[C@@H]3C[C@]5([C@H]([C@H]14)CC[C@@]56CCC(O6)=O)C)C)=O)=O	
123	XCT790	725247-18-7	COC1=C(OCC2=C(C(F)(F)F)C=C(C(F)(F)F)C=C2)C=CC(/C=C(C(NC3=NN=C(C(F)(F)F)S3)=O)\C#N)=C1	
124	Ormeloxifene	31477-60-8	CC1(C)[C@H](C2=CC=CC=C2)[C@@H](C3=CC=C(OCCN4CCCC4)C=C3)C5=C(C=C(OC)C=C5)O1	
125	Mometasone furoate	83919-23-7	C[C@@H]1C[C@H]2[C@@H]3CCC4=CC(C=C[C@]4(C)[C@@]3(Cl)[C@@H](O)C[C@]2(C)[C@@]1(OC(C5=CC=CO5)=O)C(CCl)=O)=O	
126	Desoximetasone	382-67-2	C[C@@H]1C[C@H]2[C@@H]3CCC4=CC(C=C[C@@]4([C@]3([C@H](C[C@@]2([C@H]1C(CO)=O)C)O)F)C)=O	
127	AZD9496	1639042-08-2	C[C@@H]1CC2=C(NC3=CC=CC=C23)[C@@H](C4=C(F)C=C(/C=C/C(O)=O)C=C4F)N1CC(C)(F)C	
128	(*S*)-Zearalenone	17924-92-4	C[C@H]1CCCC(CCC/C=C/C2=C(C(O1)=O)C(O)=CC(O)=C2)=O	
129	(*S*)-Zearalanone	5975-78-0	C[C@H]1CCCC(CCCCCC2=C(C(O1)=O)C(O)=CC(O)=C2)=O	
130	*β*-Zearalanol	42422-68-4	C[C@H]1CCC[C@@H](O)CCCCCC2=C(C(O1)=O)C(O)=CC(O)=C2	
131	*α*-Zearalanol	26538-44-3	C[C@H]1CCC[C@H](O)CCCCCC2=C(C(O1)=O)C(O)=CC(O)=C2	

No.	CG compound candidate	CAS-No.	Smiles	Reason for exclusion
132	Beclomethasone	4419-39-0	C[C@H]1C[C@H]2[C@@H]3CCC4=CC(C=C[C@]4(C)[C@@]3(Cl)[C@@H](O)C[C@]2(C)[C@@]1(O)C(CO)=O)=O	
133	Betamethasone	378-44-9	C[C@H]1C[C@H]2[C@@H]3CCC4=CC(C=C[C@@]4([C@]3([C@H](C[C@@]2([C@]1(C(CO)=O)O)C)O)F)C)=O	
134	Methylprednisolone	83-43-2	C[C@H]1C[C@H]2[C@@H]3CC[C@](C(CO)=O)([C@]3(C[C@@H]([C@@H]2[C@]4(C=CC(C=C14)=O)C)O)C)O	
135	Ostarine	841205-47-8	C[C@](C(NC1=CC(C(F)(F)F)=C(C#N)C=C1)=O)(O)COC2=CC=C(C#N)C=C2	
136	S-23	1010396-29-8	C[C@](C(NC1=CC(C(F)(F)F)=C(C#N)C=C1)=O)(O)COC2=CC(F)=C(Cl)C=C2	
137	Methyltrienolone	965-93-5	C[C@@]1(CC[C@H]2[C@@H]3CCC4=CC(CCC4=C3C=C[C@@]21C)=O)O	
138	Fluoxymesterone	76-43-7	C[C@@]1(CC[C@H]2[C@@H]3CCC4=CC(CC[C@@]4([C@]3([C@H](C[C@@]21C)O)F)C)=O)O	
139	Prednisone	53-03-2	C[C@]12CC([C@H]3[C@H]([C@@H]1CC[C@@]2(C(CO)=O)O)CCC4=CC(C=C[C@@]43C)=O)=O	
140	Aldosterone	52-39-1	C[C@]12CCC(C=C1CC[C@@H]3[C@@H]2[C@H](C[C@@]4([C@H](CC[C@@H]34)C(CO)=O)C=O)O)=O	
141	2-Hydroxyestradiol	362-05-0	C[C@]12CC[C@H]3[C@@H](CCC4=CC(O)=C(O)C=C34)[C@@H]1CC[C@@H]2O	
142	*17α*-Estradiol	57-91-0	C[C@]12CC[C@H]3[C@@H](CCC4=C3C=CC(O)=C4)[C@@H]1CC[C@H]2O	
143	Estetrol	15183-37-6	C[C@]12CC[C@H]3[C@@H](CCC4=C3C=CC(O)=C4)[C@@H]1[C@@H](O)[C@@H](O)[C@@H]2O	
144	Drospirenone	67392-87-4	C[C@]12CC[C@H]3[C@@H]([C@H]4C[C@H]4C5=CC(CC[C@@]53C)=O)[C@@H]1[C@@H]6C[C@@H]6[C@@]27CCC(O7)=O	
145	Corticosterone	50-22-6	C[C@]12C[C@@H]([C@H]3[C@H]([C@@H]1CC[C@@H]2C(CO)=O)CCC4=CC(CC[C@@]43C)=O)O	
146	Hydrocortisone	50-23-7	C[C@]12C[C@@H]([C@H]3[C@H]([C@@H]1CC[C@@]2(C(CO)=O)O)CCC4=CC(CC[C@@]43C)=O)O	
147	Stanozolol	10418-03-8	C[C@]12CC[C@H]3[C@@H](CC[C@@H]4[C@]3(C)CC5=C(NN=C5)C4)[C@@H]1CC[C@@]2(O)C	
148	Bazedoxifene	198481-32-2	CC1=C(C2=CC=C(O)C=C2)N(CC3=CC=C(OCCN4CCCCCC4)C=C3)C5=C1C=C(O)C=C5	
149	Methylpiperidinopyrazole	289726-02-9	CC1=C(C2=CC=C(OCCN3CCCCC3)C=C2)N(C4=CC=C(O)C=C4)N=C1C5=CC=C(O)C=C5	
150	BMS564929	627530-84-1	CC1=C(N2C(C3C(O)CCN3C2=O)=O)C=CC(C#N)=C1Cl	
151	RAD140	1182367-47-0	CC1=C(N[C@H]([C@@H](O)C)C2=NN=C(C3=CC=C(C#N)C=C3)O2)C=CC(C#N)=C1Cl	
152	Pyrvinium pamoate	3536-41-6	O=C(O)C1=CC2=CC=CC=C2C(CC3=C([O-])C(C(O)=O)=CC4=CC=CC=C34)=C1[O-]	
153	Diarylpropionitrile	1428-67-7	N#CC(C1=CC=C(O)C=C1)CC2=CC=C(O)C=C2	
154	(*R*)-Diarylpropionitrile	524047-78-7	N#C[C@@H](C1=CC=C(O)C=C1)CC2=CC=C(O)C=C2	
155	CL-278474	58125-33-0	O=C(NC1=C(C#N)C2=C(CCCC2)S1)CN3CCCCC3	
156	PF-03882845	1023650-66-9	N#CC(C=C1)=C(Cl)C=C1N2N=C3C(C=CC(C(O)=O)=C4)=C4CC[C@@H]3[C@@H]2C5CCCC5	
157	PF-998425	1076225-27-8	N#CC(C=C1)=C(C(F)(F)F)C=C1[C@@H]2[C@H](O)CCCC2	
158	Salicylamide	65-45-2	O=C(N)C1=CC=CC=C1O	

No.	CG compound candidate	CAS-No.	Smiles	Reason for exclusion
159	FERB-033	1111084-78-6	O/N=C/C(C=C1)=C(O)C(Cl)=C1C2=CC(F)=C(O)C=C2	
160	Isoliquiritigenin	961-29-5	O=C(C1=C(O)C=C(O)C=C1)/C=C/C2=CC=C(O)C=C2	
161	Phloretin	60-82-2	O=C(C1=C(O)C=C(O)C=C1O)CCC2=CC=C(O)C=C2	
162	G67882	853352-52-0	CC1=CC=CC=C1NC2=NC(COC(C3=CC=C(N(C)C)C=C3)=O)=NC(N)=N2	
163	Liquiritigenin	578-86-9	O=C1C(C=CC(O)=C2)=C2O[C@H](C3=CC=C(O)C=C3)C1	
164	Anthraflavic acid	84-60-6	OC1=CC(C(C2=C(C=C(O)C=C2)C3=O)=O)=C3C=C1	
165	GSK9027	1229096-88-1	O=S(CC1=CC=CC=C1)(NC2=CC(C(F)(F)F)=C(C3=CC4=C(N(C5=CC=C(F)C=C5)N=C4)C=C3)C=C2)=O	
166	WAY-607413	106149-18-2	O=S(NC(C=C1)=CC=C1OC2=CC=CC=C2)(C3=CC=CC=C3)=O	
167	4',5-Dihydroxyflavone	6665-67-4	O=C1C=C(C2=CC=C(O)C=C2)OC3=CC=CC(O)=C31	
168	PHTPP	805239-56-9	OC(C=C1)=CC=C1C2=C3N=C(C(F)(F)F)C=C(C(F)(F)F)N3N=C2C4=CC=CC=C4	
169	AC-8398	63676-22-2	OC(C=C1)=CC(S2)=C1C=C2C3=CC=C(O)C=C3	
170	WAY200070	440122-66-7	OC1=CC2=C(C(Br)=C1)OC(C3=CC=C(O)C=C3)=N2	
171	(*R*)-Equol	221054-79-1	OC1=CC2=C(C=C1)C[C@H](C3=CC=C(O)C=C3)CO2	
172	Erteberel	533884-09-2	OC1=CC([C@H]2[C@@H]3CCC2)=C(O[C@H]3C4=CC=C(O)C=C4)C=C1	
173	Lasofoxifene	180916-16-9	OC1=CC2=C(C=C1)[C@H]([C@@H](C3=CC=CC=C3)CC2)C(C=C4)=CC=C4OCCN5CCCC5	
174	WAY-628730	2832-97-5	N1(C2=CSC(C3=CC=CC=C3)=C2)CCOCC1	
175	VPC-14228	19983-28-9	N1(C2=NC(C3=CC=CC=C3)=CS2)CCOCC1	
176	AY-20590	57490-73-0	C12=CC=CC=C1N=C(C3=CC=CO3)C(C4=CC=CO4)=N2	

Supplementary References

1. Meijer, I. *et al.* Chemical starting matter for HNF4α ligand discovery and chemogenomics. *Int J Mol Sci* **21**, 1–11 (2020).

2. Schäfer, A. *et al.* Influence of chlorine or fluorine substitution on the estrogenic properties of 1-alkyl-2,3,5-tris(4-hydroxyphenyl)-1H-pyrroles. *J Med Chem* **55**, 9607–9618 (2012).

3. Covaleda, A. M. S. *et al.* Influence of cellular ERα/ERβ ratio on the ERα-agonist induced proliferation of human T47D breast cancer cells. *Toxicol Sci* **105**, 303–311 (2008).

4. De Savi, C. *et al.* Optimization of a Novel Binding Motif to (E)-3-(3,5-Difluoro-4-((1R,3R)-2-(2-fluoro-2-methylpropyl)-3-methyl-2,3,4,9-tetrahydro-1H-pyrido[3,4-b]indol-1-yl)phenyl)acrylic Acid (AZD9496), a Potent and Orally Bioavailable Selective Estrogen Receptor Downregulator and Antagonist. *J Med Chem* **58**, 8128–8140 (2015).

5. Christiansen, L. B. *et al.* Synthesis and Biological Evaluation of Novel Thio-Substituted Chromanes as High-Affinity Partial Agonists for the Estrogen Receptor. *Bioorg Med Chem Lett* **12**, 17–19 (2002).

6. Burks, H. E. *et al.* Discovery of an Acrylic Acid Based Tetrahydroisoquinoline as an Orally Bioavailable Selective Estrogen Receptor Degrader for ERα+ Breast Cancer. *J Med Chem* **60**, 2790–2818 (2017).

7. Tria, G. S. *et al.* Discovery of LSZ102, a potent, orally bioavailable selective estrogen receptor degrader (SERD) for the treatment of estrogen receptor positive breast cancer. *J Med Chem* **61**, 2837–2864 (2018).

8. Roll, D. M. *et al.* The lecanindoles, nonsteroidal progestins from the terrestrial fungus Verticillium lecanii 6144. *J Nat Prod* **72**, 1944–1948 (2009).

9. Yu, D. D., Lin, W., Chen, T. & Forman, B. M. Development of time resolved fluorescence resonance energy transfer-based assay for FXR antagonist discovery. *Bioorg Med Chem* **21**, 4266–4278 (2013).

10. Morisseau, C., Pakhomova, S., Hwang, S. H., Newcomer, M. E. & Hammock, B. D. Inhibition of soluble epoxide hydrolase by fulvestrant and sulfoxides. *Bioorg Med Chem Lett* **23**, 3818–3821 (2013).

11. Meyers, M. J., Jun, S., Carlson, K. E., Katzenellenbogen, B. S. & Katzenellenbogen, J. A. Estrogen receptor subtype-selective ligands: Asymmetric synthesis and biological evaluation of cis- and trans-5,11-dialkyl-5,6,11,12- tetrahydrochrysenes. *J Med Chem* **42**, 2456–2468 (1999).

12. Sun, J. *et al.* Novel Ligands that Function as Selective Estrogens or Antiestrogens for Estrogen Receptor-α or Estrogen Receptor-β*. *Endocrinology* **140**, 800–804 (1999).

13. Lai, A. *et al.* Identification of GDC-0810 (ARN-810), an Orally Bioavailable Selective Estrogen Receptor Degrader (SERD) that Demonstrates Robust Activity in Tamoxifen-Resistant Breast Cancer Xenografts. *J Med Chem* **58**, 4888–4904 (2015).

14. Waibel, M. *et al.* Bibenzyl- and stilbene-core compounds with non-polar linker atom substituents as selective ligands for estrogen receptor beta. *Eur J Med Chem* **44**, 3412–3424 (2009).

15. Meyers, M. J. *et al.* Estrogen receptor-β potency-selective ligands: Structure-activity relationship studies of diarylpropionitriles and their acetylene and polar analogues. *J Med Chem* **44**, 4230–4251 (2001).

16. Sedlák, D., Novák, P., Kotora, M. & Bartůněk, P. Synthesis and evaluation of 17α-Arylestradiols as ligands for estrogen receptor α and β. *J Med Chem* **53**, 4290–4294 (2010).

17. Lin, W., Yang, L., Chai, S. C., Lu, Y. & Chen, T. Development of CINPA1 analogs as novel and potent inverse agonists of constitutive androstane receptor. *Eur J Med Chem* **108**, 505–528 (2016).

18. Pryde, D. C. *et al.* Aldehyde oxidase: An enzyme of emerging importance in drug discovery. *J Med Chem* **53**, 8441–8460 (2010).

19. Fontana, E., Dansette, P. M. & Poli, S. M. Cytochrome P450 Enzymes Mechanism Based Inhibitors: Common Sub-Structures and Reactivity. *Curr Drug Metab* **6**, 413–454 (2005).

20. Rohn, K. J., Cook, I. T., Leyh, T. S., Kadlubar, S. A. & Falany, C. N. Potent Inhibition of Human Sulfotransferase 1A1 by17α-Ethinylestradiol: Role of 3'-Phosphoadenosine 5'-Phosphosulfate Binding and Structural Rearrangements inRegulating Inhibition and Activity. *Drug Metab Dispos* **40**, 1588–1595 (2012).

21. Wittwer, M. B. *et al.* Discovery of potent, selective multidrug and toxin extrusion transporter 1 (MATE1, SLC47A1) inhibitors through prescription drug profiling and computational modeling. *J Med Chem* **56**, 781–795 (2013).

22. Komm, B. S. *et al.* Bazedoxifene acetate: A selective estrogen receptor modulator with improved selectivity. *Endocrinology* **146**, 3999–4008 (2005).

23. Luan, F., Liu, H. T., Ma, W. P. & Fan, B. T. Classification of estrogen receptor-β ligands on the basis of their binding affinities using support vector machine and linear discriminant analysis. *Eur J Med Chem* **43**, 43–52 (2008).

24. Malamas, M. S. *et al.* Design and synthesis of aryl diphenolic azoles as potent and selective estrogen receptor-β ligands. *J Med Chem* **47**, 5021–5040 (2004).

25. Compton, D. R. *et al.* Pyrazolo[1,5-α]pyrimidines: Estrogen receptor ligands possessing estrogen receptor β antagonist activity. *J Med Chem* **47**, 5872–5893 (2004).

26. Sun, Y. C. *et al.* Estrogenic activities of isoflavones and flavones and their structure-activity relationships. *Planta Med* **74**, 25–32 (2008).

27. Vuorinen, A. *et al.* Potential Antiosteoporotic Natural Product Lead Compounds That Inhibit 17β-Hydroxysteroid Dehydrogenase Type 2. *J Nat Prod* **80**, 965–974 (2017).

28. Huang, J. *et al.* Development of benzoxazole deoxybenzoin oxime and acyloxylamine derivatives targeting innate immune sensors and xanthine oxidase for treatment of gout. *Bioorg Med Chem* **26**, 1653–1664 (2018).

29. Shim, S. H. 20S proteasome inhibitory activity of flavonoids isolated from Spatholobus suberectus. *Phytotherapy Research* **25**, 615–618 (2011).

30. Suetsugi, M., Su, L., Karlsberg, K., Yuan, Y.-C. & Chen, S. Flavone and Isoflavone Phytoestrogens Are Agonists of Estrogen-Related Receptors. *Mol Cancer Res* **1**, 981–991 (2003).

31. Karioti, A., Ceruso, M., Carta, F., Bilia, A. R. & Supuran, C. T. New natural product carbonic anhydrase inhibitors incorporating phenol moieties. *Bioorg Med Chem* **23**, 7219–7225 (2015).

32. Zimmermann, T. J. *et al.* Discovery of a potent and selective inhibitor for human carbonyl reductase 1 from propionate scanning applied to the macrolide zearalenone. *Bioorg Med Chem* **17**, 530–536 (2009).

33. Dutour, R. & Poirier, D. Inhibitors of cytochrome P450 (CYP) 1B1. *Eur J Med Chem* **135**, 296–306 (2017).

34. Schuster, D. *et al.* Discovery of nonsteroidal 17β-hydroxysteroid dehydrogenase 1 inhibitors by pharmacophore-based screening of virtual compound libraries. *J Med Chem* **51**, 4188–4199 (2008).

35. Le Lain, R., Nicholls, P. J., Smith, H. J. & Maharlouie, F. H. Inhibitors of human and rat testes microsomal 17 β-hydroxysteriod dehydrogenase (17-β-HSD) as potential agents for prostatic cancer. *J Enzyme Inhib* **16**, 35–45 (2001).

36. Yu, D. D., Huss, J. M., Li, H. & Forman, B. M. Identification of novel inverse agonists of estrogen-related receptors ERRγ and ERRβ. *Bioorg Med Chem* **25**, 1585–1599 (2017).

37. Knox, A. *et al.* Development of bivalent triarylalkene- and cyclofenil-derived dual estrogen receptor antagonists and downregulators. *Eur J Med Chem* **192**, (2020).

38. Scott, S. A. *et al.* Discovery of desketoraloxifene analogues as inhibitors of mammalian, pseudomonas aeruginosa, and NAPE phospholipase D enzymes. *ACS Chem Biol* **10**, 421–432 (2015).

39. Zuercher, W. J. *et al.* Identification and structure-activity relationship of phenolic acyl hydrazones as selective agonists for the estrogen-related orphan nuclear receptors ERRβ and ERRγ. *J Med Chem* **48**, 3107–3109 (2005).

40. Schopfer, U. *et al.* Toward selective ERβ agonists for central nervous system disorders: Synthesis and characterization of aryl benzthiophenes. *J Med Chem* **45**, 1399–1401 (2002).

41. Xu, S. *et al.* 1-Phenyl-4-benzoyl-1H-1,2,3-triazoles as orally bioavailable transcriptional function suppressors of Estrogen-related receptor α. *J Med Chem* **56**, 4631–4640 (2013).

42. Peng, L. *et al.* Identification of New Small-Molecule Inducers of Estrogen-related Receptor α (ERRα) Degradation. *ACS Med Chem Lett* **10**, 767–772 (2019).

43. Patch, R. J. *et al.* Identification of diaryl ether-based ligands for estrogen-related receptor α as potential antidiabetic agents. *J Med Chem* **54**, 788–808 (2011).

44. Busch, B. B. *et al.* Identification of a selective inverse agonist for the orphan nuclear receptor estrogen-related receptor α. *J Med Chem* **47**, 5593–5596 (2004).

45. Lin, H. *et al.* Design, synthesis, and evaluation of simple phenol amides as ERRγ agonists. *Bioorg Med Chem Lett* **28**, 1313–1319 (2018).

46. Zuercher, W. J. *et al.* Identification and structure-activity relationship of phenolic acyl hydrazones as selective agonists for the estrogen-related orphan nuclear receptors ERRβ and ERRγ. *J Med Chem* **48**, 3107–3109 (2005).

47. Yu, D. D. & Forman, B. M. Identification of an agonist ligand for estrogen-related receptors ERRβ/γ. *Bioorg Med Chem Lett* **15**, 1311–1313 (2005).

48. Chao, E. Y. H. *et al.* Structure-guided synthesis of tamoxifen analogs with improved selectivity for the orphan ERRγ. *Bioorg Med Chem Lett* **16**, 821–824 (2006).

49. Matsushima, A. *et al.* ERRγ tethers strongly bisphenol A and 4-α-cumylphenol in an induced-fit manner. *Biochem Biophys Res Commun* **373**, 408–413 (2008).

50. Liu, X. *et al.* Bisphenol AF: Halogen bonding effect is a major driving force for the dual ERα-agonist and ERβ-antagonist activities. *Bioorg Med Chem* **28**, (2020).

51. Maruyama, K. *et al.* Structure-activity relationships of bisphenol a analogs at estrogen receptors (ERs): Discovery of an ERα-selective antagonist. *Bioorg Med Chem Lett* **23**, 4031–4036 (2013).

52. Yates, C. M. *et al.* Structure guided design of 5-arylindazole glucocorticoid receptor agonists and antagonists. *J Med Chem* **53**, 4531–4544 (2010).

53. Hemmerling, M. *et al.* Selective Nonsteroidal Glucocorticoid Receptor Modulators for the Inhaled Treatment of Pulmonary Diseases. *J Med Chem* **60**, 8591–8605 (2017).

54. Werkström, V., Prothon, S., Ekholm, E., Jorup, C. & Edsbäcker, S. Safety, Pharmacokinetics and Pharmacodynamics of the Selective Glucocorticoid Receptor Modulator AZD5423 after Inhalation in Healthy Volunteers. *Basic Clin Pharmacol Toxicol* **119**, 574–581 (2016).

55. Auerbach, S. S. DrugMatrix in vitro pharmacology data. *National Toxicology Program: Dept of Health and Human Services* https://www.guidetopharmacology.org/GRAC/LigandDisplayForward?tab=biology&ligandId=7059.

56. Schäcke, H. *et al.* Characterization of ZK 245186, a novel, selective glucocorticoid receptor agonist for the topical treatment of inflammatory skin diseases. *Br J Pharmacol* **158**, 1088–1103 (2009).

57. Rupprecht, R. *et al.* Pharmacological and functional characterization of human mineralocorticoid and glucocorticoid receptor ligands. *Eur J Pharmacol* **247**, 145 (1993).

58. Millan, D. S. *et al.* Design and synthesis of long acting inhaled corticosteroids for the treatment of asthma. *Bioorg Med Chem Lett* **21**, 5826–5830 (2011).

59. Hemmerling, M. *et al.* Selective Nonsteroidal Glucocorticoid Receptor Modulators for the Inhaled Treatment of Pulmonary Diseases. *J Med Chem* **60**, 8591–8605 (2017).

60. Kern, J. C. *et al.* 1-Methyl-1H-pyrrole-2-carbonitrile containing tetrahydronaphthalene derivatives as non-steroidal progesterone receptor antagonists. *Bioorg Med Chem Lett* **20**, 4816–4818 (2010).

61. Zhang, P. *et al.* 6-Aryl-1,4-dihydro-benzo[d][1,3]oxazin-2-ones: A novel class of potent, selective, and orally active nonsteroidal progesterone receptor antagonists. *J Med Chem* **45**, 4379–4382 (2002).

62. Hamann, L. G. *et al.* Synthesis and Biological Activity of Novel Nonsteroidal Progesterone Receptor Antagonists Based on Cyclocymopol Monomethyl Ether. *J Med Chem* **39**, 1778–1789 (1996).

63. Kern, J. C. *et al.* 1,5-Dihydro-benzo[e][1,4]oxazepin-2(1H)-ones containing a 7-(5′-cyanopyrrol-2-yl) group as nonsteroidal progesterone receptor modulators. *Bioorg Med Chem Lett* **18**, 5015–5017 (2008).

64. Zhang, P. *et al.* 7-Aryl 1,5-dihydro-benzo[e][1,4]oxazepin-2-ones and analogs as non-steroidal progesterone receptor antagonists. *Bioorg Med Chem* **16**, 6589–6600 (2008).

65. Zhi, L. *et al.* Development of progesterone receptor antagonists from 1,2-dihydrochromeno[3,4-f]quinoline agonist pharmacophore. *Bioorg Med Chem Lett* **13**, 2075–2078 (2003).

66. Rew, Y. *et al.* Discovery of a Potent and Selective Steroidal Glucocorticoid Receptor Antagonist (ORIC-101). *J Med Chem* **61**, 7767–7784 (2018).

67. Du, X. *et al.* Discovery of a potent steroidal glucocorticoid receptor antagonist with enhanced selectivity against the progesterone and androgen receptors (OP-3633). *J Med Chem* **62**, 6751–6764 (2019).

68. Morgan, R. E. *et al.* A Multifactorial Approach to Hepatobiliary Transporter Assessment Enables Improved Therapeutic Compound Development. *Toxicol Sci* **136**, 216–241 (2013).

69. Saeed, A. *et al.* 2-Chloro-4-[[(1R,2R)-2-hydroxy-2-methyl-cyclopentyl]amino]-3-methyl-benzonitrile: A Transdermal Selective Androgen Receptor Modulator (SARM) for Muscle Atrophy. *J Med Chem* **59**, 750–755 (2016).

70. Zhi, L. *et al.* 5-Alkyl 1,2-dihydrochromeno[3,4-f]quinolines: A novel class of nonsteroidal progesterone receptor modulators. *Bioorg Med Chem Lett* **8**, 3365–3370 (1998).

71. Zhi, L., Tegley, C. M., Marschke, K. B., Mais, D. E. & Jones, T. K. 5-aryl-1,2,3,4-tetrahydrochromeno[3,4-f]quinolin-3-ones as a novel class of nonsteroidal progesterone receptor agonists: Effect of A-ring modification. *J Med Chem* **42**, 1466–1472 (1999).

72. Edwards, J. P. *et al.* 5-Aryl-1,2-dihydro-5H-chromeno[3,4-f]quinolines as Potent, Orally Active, Nonsteroidal Progesterone Receptor Agonists: The Effect of D-Ring Substituents. *J Med Chem* **41**, 303–310 (1998).

73. Fensome, A. *et al.* Novel 5-aryl-1,3-dihydro-indole-2-thiones: Potent, orally active progesterone receptor agonists. *Bioorg Med Chem Lett* **13**, 1317–1320 (2003).

74. Zhi, L. *et al.* 5-Aryl-1,2-dihydrochromeno[3,4-f]quinolines: A Novel Class of Nonsteroidal Human Progesterone Receptor Agonists. *J Med Chem* **41**, 291–302 (1998).

75. Tegley, C. M. *et al.* 5-benzylidene 1,2-dihydrochromeno[3,4-f]quinolines, a novel class of nonsteroidal human progesterone receptor agonists. *J Med Chem* **41**, 4354–4359 (1998).

76. Zhi, L. *et al.* 5-Benzylidene-1,2-dihydrochromeno[3,4-f]quinolines as selective progesterone receptor modulators. *J Med Chem* **46**, 4104–4112 (2003).

77. Gazvoda, M. *et al.* 2,3-Diarylpropenoic acids as selective non-steroidal inhibitors of type-5 17β-hydroxysteroid dehydrogenase (AKR1C3). *Eur J Med Chem* **62**, 89–97 (2013).

78. Eignerová, B., Sedlák, D., Dračínský, M., Bartůněk, P. & Kotora, M. Synthesis and biochemical characterization of a series of 17α-perfluoroalkylated estradiols as selective ligands for estrogen receptor α. *J Med Chem* **53**, 6947–6953 (2010).

79. Meyers, M. J. *et al.* Discovery of (3 S,3a R)-2-(3-chloro-4-cyanophenyl)-3-cyclopentyl-3,3a,4,5- tetrahydro-2 H -benzo[g]indazole-7-carboxylic acid (PF-3882845), an orally efficacious mineralocorticoid receptor (MR) antagonist for hypertension and nephropathy. *J Med Chem* **53**, 5979–6002 (2010).

80. Arhancet, G. B. *et al.* Discovery of novel cyanodihydropyridines as potent mineralocorticoid receptor antagonists. *J Med Chem* **53**, 5970–5978 (2010).

81. Casimiro-Garcia, A. *et al.* Identification of (R)-6-(1-(4-cyano-3-methylphenyl)-5-cyclopentyl-4,5- dihydro-1H-pyrazol-3-yl)-2-methoxynicotinic acid, a highly potent and selective nonsteroidal mineralocorticoid receptor antagonist. *J Med Chem* **57**, 4273–4288 (2014).

82. Meyer, U., Costantino, G., Macchiarulo, A. & Pellicciari, R. Is antagonism of E/Z-guggulsterone at the farnesoid X receptor mediated by a noncanonical binding site? A molecular modeling study. *J Med Chem* **48**, 6948–6955 (2005).

83. Rewinkel, J. *et al.* 11-(Pyridinylphenyl)steroids-A new class of mixed-profile progesterone agonists/antagonists. *Bioorg Med Chem* **16**, 2753–2763 (2008).

84. Mohler, M. L. *et al.* Nonsteroidal Selective Androgen Receptor Modulators (SARMs): Dissociating the anabolic and androgenic activities of the androgen receptor for therapeutic benefit. *J Med Chem* **52**, 3597–3617 (2009).

85. Yin, D. *et al.* Pharmacodynamics of selective androgen receptor modulators. *J Pharmacol Exp Ther* **304**, 1334–1340 (2003).

86. Marhefka, C. A. *et al.* Design, Synthesis, and Biological Characterization of Metabolically Stable Selective Androgen Receptor Modulators. *J Med Chem* **47**, 993–998 (2004).

87. Jie, J. L. *et al.* Rational design and synthesis of 4-((1R,2R)-2-hydroxycyclohexyl)-2(trifluoromethyl)benzonitrile (PF-998425), a novel, nonsteroidal androgen receptor antagonist devoid of phototoxicity for dermatological indications. *J Med Chem* **51**, 7010–7014 (2008).

88. Hamann, L. G. *et al.* Synthesis and Biological Activity of a Novel Series of Nonsteroidal, Peripherally Selective Androgen Receptor Antagonists Derived from 1,2-Dihydropyridono[5,6-g]quinolines. *J Med Chem* **41**, 623–639 (1998).

89. Ferla, S. *et al.* Rational design and synthesis of novel anti-prostate cancer agents bearing a 3,5-bis-trifluoromethylphenyl moiety. *Bioorg Med Chem Lett* **26**, 3636–3640 (2016).

90. Li, J. J. *et al.* Discovery of potent and muscle selective androgen receptor modulators through scaffold modifications. *J Med Chem* **50**, 3015–3025 (2007).

91. Hamann, L. G. *et al.* Tandem optimization of target activity and elimination of mutagenic potential in a potent series of N-aryl bicyclic hydantoin-based selective androgen receptor modulators. *Bioorg Med Chem Lett* **17**, 1860–1864 (2007).

92. Isigkeit, L., Chaikuad, A. & Merk, D. A Consensus Compound/Bioactivity Dataset for Data-Driven Drug Design and Chemogenomics. *Molecules* **27**, (2022).
