# Supplementary material for: Chemogenomics for steroid hormone receptors (NR3)
Source: Commun Chem. 2025 Feb 3;8:29. doi: 10.1038/s42004-025-01427-z (PMC11790914; doi:10.1038/s42004-025-01427-z)
Supplement: Supplementary file 3 — Description of Additional Supplementary Files [file 42004_2025_1427_MOESM3_ESM.pdf]

Description of Additional Supplementary Files

File name: Supplementary Data 1

Description: Source data underlying the Figures 2-6 and the Supplementary Figures.
