# Supplementary material for: Chemogenomics for steroid hormone receptors (NR3)
Source: Commun Chem. 2025 Feb 3;8:29. doi: 10.1038/s42004-025-01427-z (PMC11790914; doi:10.1038/s42004-025-01427-z)
Supplement: Supplementary file 5 — reporting summary [file 42004_2025_1427_MOESM5_ESM.pdf]

nature portfolio

Corresponding author(s): Daniel Merk

Last updated by author(s): Jan 20, 2025

Reporting Summary

Nature Portfolio wishes to improve the reproducibility of the work that we publish. This form provides structure for consistency and transparency in reporting. For further information on Nature Portfolio policies, see our Editorial Policies and the Editorial Policy Checklist.

Statistics

For all statistical analyses, confirm that the following items are present in the figure legend, table legend, main text, or Methods section.

n/a	Confirmed	
☐	☒	The exact sample size (*n*) for each experimental group/condition, given as a discrete number and unit of measurement
☐	☒	A statement on whether measurements were taken from distinct samples or whether the same sample was measured repeatedly
☐	☒	The statistical test(s) used AND whether they are one- or two-sided<br>*Only common tests should be described solely by name; describe more complex techniques in the Methods section.*
☒	☐	A description of all covariates tested
☒	☐	A description of any assumptions or corrections, such as tests of normality and adjustment for multiple comparisons
☒	☐	A full description of the statistical parameters including central tendency (e.g. means) or other basic estimates (e.g. regression coefficient) AND variation (e.g. standard deviation) or associated estimates of uncertainty (e.g. confidence intervals)
☒	☐	For null hypothesis testing, the test statistic (e.g. *F*, *t*, *r*) with confidence intervals, effect sizes, degrees of freedom and *P* value noted<br>*Give P values as exact values whenever suitable.*
☒	☐	For Bayesian analysis, information on the choice of priors and Markov chain Monte Carlo settings
☒	☐	For hierarchical and complex designs, identification of the appropriate level for tests and full reporting of outcomes
☒	☐	Estimates of effect sizes (e.g. Cohen's *d*, Pearson's *r*), indicating how they were calculated

Our web collection on statistics for biologists contains articles on many of the points above.

Software and code

Policy information about availability of computer code

Data collection	Compound-bioactivity data were collected from public resources (ChEMBL, PubChem, IUPHAR/BPS, BindingDB, Probes&Drugs) and combined using KNIME (v4.5) with rdkit software (version 2022.09.1).
Data analysis	Data were processed and analyzed using KNIME (version 4.5), Python (version 3.8), Python module scikit-learn (version 1.3.0), rdkit (version 2022.09.1), GraphPad (version 9.5.1), Thermal Shift Software (version 1.4).

For manuscripts utilizing custom algorithms or software that are central to the research but not yet described in published literature, software must be made available to editors and reviewers. We strongly encourage code deposition in a community repository (e.g. GitHub). See the Nature Portfolio guidelines for submitting code & software for further information.

Data

Policy information about availability of data

All manuscripts must include a data availability statement. This statement should provide the following information, where applicable:

- Accession codes, unique identifiers, or web links for publicly available datasets
- A description of any restrictions on data availability
- For clinical datasets or third party data, please ensure that the statement adheres to our policy

All data supporting the results of this study are available in the Supplementary Information. Source data are provided with this paper in Supplementary Data 1

Human research participants

Policy information about studies involving human research participants and Sex and Gender in Research.

Reporting on sex and gender	n/a
Population characteristics	n/a
Recruitment	n/a
Ethics oversight	n/a

Note that full information on the approval of the study protocol must also be provided in the manuscript.

Field-specific reporting

Please select the one below that is the best fit for your research. If you are not sure, read the appropriate sections before making your selection.

☒ Life sciences ☐ Behavioural & social sciences ☐ Ecological, evolutionary & environmental sciences

For a reference copy of the document with all sections, see nature.com/documents/nr-reporting-summary-flat.pdf

Life sciences study design

All studies must disclose on these points even when the disclosure is negative.

Sample size	n/a (the study includes no in vivo experiments)
Data exclusions	No data have been excluded
Replication	All in vitro biological experiments have been successfully replicated in at least three biologically independent repeats.
Randomization	Not relevant in this study. Only in vitro assays were conducted in which samples are derived from the same clone and in which the entire CG set was applied. Nevertheless, different repeats were conducted on different dates and from different harvests/transfections/seeds to account for covariates introduced by the experimental procedures
Blinding	Investigators were blinded in experiments in which the complete CG library was applied/used as a set. Additionally, the biological proof-of-concept applications of the CG set were purely descriptive and not intended to provide evidence for an a priori hypothesis. Blinding was not meaningful in experiments referring to analytical characterization, quality control and selectivity profiling.

Reporting for specific materials, systems and methods

We require information from authors about some types of materials, experimental systems and methods used in many studies. Here, indicate whether each material, system or method listed is relevant to your study. If you are not sure if a list item applies to your research, read the appropriate section before selecting a response.

Materials & experimental systems

n/a	Involved in the study	
☒	☐	Antibodies
☐	☒	Eukaryotic cell lines
☒	☐	Palaeontology and archaeology
☒	☐	Animals and other organisms
☒	☐	Clinical data
☒	☐	Dual use research of concern

Methods

n/a	Involved in the study	
☒	☐	ChIP-seq
☒	☐	Flow cytometry
☒	☐	MRI-based neuroimaging

Eukaryotic cell lines

Policy information about cell lines and Sex and Gender in Research

Cell line source(s)	HEK293T (ATCC, CRL-1573) were obtained from ATCC. HEK293 cells containing a GFP reporter gene for NF-kB activity

Cell line source(s)	(#14-700ACL) and Hela cells containing a Renilla luciferase reporter gene for ATF6 activity (#14-138ACL) were obtained from Abeomics Inc. (San Diego, California, USA).
Authentication	Cells were obtained from above-described sources and not further authenticated.
Mycoplasma contamination	Cell lines have been monitored/tested negative for mycoplasma contamination.
Commonly misidentified lines (See ICLAC register)	No commonly misidentified cell lines were used in the study.
